# Revealing Invisible Beauty, Ultra Detailed: The Influence of Low Cost UV Exposure on Natural History Specimens in 2D+ Digitization

**DOI:** 10.1371/journal.pone.0161572

**Published:** 2016-08-18

**Authors:** Jonathan Brecko, Aurore Mathys, Wouter Dekoninck, Marleen De Ceukelaire, Didier VandenSpiegel, Patrick Semal

**Affiliations:** 1 Scientific Heritage Service, Royal Belgian Institute of Natural Sciences, Vautierstraat 29, B-1000 Brussels, Belgium; 2 Biological Collection and Data Management, Royal Museum for Central Africa, Leuvensesteenweg 13, B-3080 Tervuren, Belgium; United States Department of Agriculture, UNITED STATES

## Abstract

Digitization of the natural history specimens usually occurs by taking detailed pictures from different sides or producing 3D models. Additionally this is normally limited to imaging the specimen while exposed by light of the visual spectrum. However many specimens can see in or react to other spectra as well. Fluorescence is a well known reaction to the ultraviolet (UV) spectrum by animals, plants, minerals etc. but rarely taken into account while examining natural history specimens. Our tests show that museum specimens still fluoresce when exposed to UV light of 395 nm and 365 nm, even after many years of preservation. When the UV exposure is used in the digitization of specimens using our low cost focus stacking (2D+) setup, the resulting pictures reveal more detail than the conventional 2D+ images. Differences in fluorescence using 395 nm or 365 nm UV lights were noticed, however there isn’t a preferred wavelength as some specimens react more to the first, while others have better results with the latter exposure. Given the increased detail and the low cost of the system, UV exposure should be considered while digitizing natural history museum collections.

## Introduction

In most digitization projects researchers try to document and duplicate collection specimens in a way as humans observe them. As an end result of the digitization process, the eventually produced digitized form has to show as much detail as possible and give an accurate representation of the original specimen. But the manner in which a certain specimen is perceived might not be the most accurate one. Human sight is not perfect at all as we can’t see in the ultraviolet, nor the infrared spectrum. Fortunately, most researchers are aware of the fact that the sight of many creatures is more sensitive to one of these spectra and do take this into account in their research ([1], [2], [3], etc).

Therefore it should withal be considered to expose our precious collection to other light sources than visible light while digitizing. Many specimens fluoresce when exposed to UV light, of which scorpions are one of the best known examples. What we see is the reaction of the particular tissues of the specimen to the UV light. The use of UV light in research isn’t new. Several scientists have shown the potential of using this light source to reveal cryptic morphologies. Volschenk [4], proposed using UV light to look at the surface morphology of scorpions. Yang *et al*. [5] and Loria & Prendini [6], showed that UV light enhances the visibility of minor ocelli in scorpions as they don’t lit up the same way as tubercles on the cuticle. Others use it to explain how animals see the world and find appropriate food resources ([7], [3]) or have found out that sexes look different from each other in UV light [8].

Because a lot of specimens fluoresce under UV exposure (Spiders: [9], [10]; Insects: [11], [12]; Crustaceans: [13]; Birds: [2], [14]; Fish: [15]; Fossils: [16], [17]; Plants: [18], [19], [20]; etc.), the possibilities which low cost UV lights have not only in digitization, but also in general for taxonomic, morphologic and other research are endless. The focus of this study will be on the use of UV light in combination with a low cost focus stacking setup, as the latter already gives highly detailed information [21]. By using these two techniques together is expected that species will reveal details, morphologies and patterns which otherwise would have remained hidden for our eyes. The scope of this paper is not to discuss why certain specimens do lit up in UV and others might not, as this has already been done in the past [10] [12]. But to show that the small extra effort of using UV light in digitization or other research might open doors for future researchers or show apparent differences, clearly visible between closely related, cryptic species, the two sexes of species etc…

The ultra violet light spectrum has wavelengths between 100 nm to 400 nm. Two wavelengths in the UV-A region (315 nm to 400 nm), 365 nm and 395 nm respectively will be tested. The reasons to test these two wavelengths is that regular black lights, which are easy to come by, generally have wavelength of 395 nm. Volschenk [4] and Holovachov [22] on the other hand suggested that several animals show more fluorescence at 365 nm. Therefore the effects of the latter wavelength, which is used in the manicure industry, will be tested as well.

To sum up the main goals of this manuscript are

- to see if UV fluorescence by natural history specimens still occurs after being stored in a (dry or wet) collection over several decades.
- to analyse whether the combination of the UV light source and the focus stacking technique will show more details than using visible light.
- to check which of the tested wavelengths will produce the best results.
- to enhance the results by applying external filters are removing internal ones.

## Materials and Methods

### The setup

For the experiments 2 different light sources, which are low cost and easy to obtain, are used. One of the light sources used consists of four 365 nm UV lights of 9W each and is normally used in the manicure industry. These lights emit a bright white-blue light. The other light bulbs were regular UV black lights of 395 nm, which emit blue light. To have an evenly lit subject, 2 lights of 395 nm are used while lighting the specimen. However, the 365 nm setup comes standard with 4 light bulbs. In all the tests these 4 lights were used, 2 on the left and 2 on the right side of a specimen. But as they produce a lot of light, a test is made as well while only 2 of the 4 lamps are lit. If the results are the same, than 2 light bulbs can be used as spare ones. To evaluate the effect of the UV lights, pictures of the specimens are taken using the same setup, but with regular flashes (non-UV).

The UV lights were mounted within a similar focus stacking set-up (2D+) as discussed in Brecko et al. [21]. The set-up consists of a Canon EOS 700D, a Canon MP-E 65 mm 1:2.8 1-5x Macro Photo Lens or a Canon EF-S 60 mm f/2.8 Macro USM, 2 Yongnuo YN-560II flash lights, one remote to control the flash lights, a Cognisys Stackshot and an Ikea Metod Kitchen cupboard.

Pictures of the specimens exposed to UV light or flashes or both, were taken, to understand the effect of the light source on the level of detail visible (Table 1). Besides the different light sources used, a yellow filter was used in combination with the different UV light sources, to block the blue color of the UV lights. The camera used still had its internal filters, so the recordings visualize merely the UV induced fluorescence of the specimens. Therefore a test with a de-filtered camera, of which the UV filter is removed, is performed as well. In this way UV rays can reach the sensor of the camera and perhaps more details can be seen.

**Table 1:** Overview of the different variables compared to each other. In the table it is clear of which type of collection a specimen is chosen to test the differences between the variables.

	Comparison	
Collection	Variable 1	Variable 2
Wet & Dry	Flash (Non-UV)	395 nm (UV)
Wet & Dry	Flash (Non-UV)	365 nm (UV)
Wet	LED	365 nm (UV)
Wet	395 nm (UV)	365 nm (UV)
Wet	4 bulbs of 365 nm (UV)	2 bulbs of 365 nm (UV)
Wet	Flash or 395 nm (UV)	Flash + 395 nm (UV)
Wet	Yellow filter + 395 nm or 365 nm (UV)	395 nm or 365 nm (UV)
Wet	De-filtered DSLR + 365 nm (UV)	Default DSLR + 365 nm (UV)

While the chosen shutter speed for the pictures lit by flash light always is 1/100s, the exposure under UV was a lot longer, depending on the light source and specimen, 1/10s up to 0.6s for the 365 nm UV lights and 5 up to 13s for the exposure with the 2 UV lights of 395 nm.

Several of the images are converted into grayscale, after auto correction for levels and contrast, to judge whether the characters are more visible due to color difference or gain in contrast and sharpness.

### The specimens

Specimens of both the dry and the wet collection (ethanol based) are used in the experiments. The largest part of dry collections in a natural history museum consist of rock material (minerals, fossils, …) or dried animals or animal parts. The dry collection specimens used in the comparisons are a hyalite mineral (RB8315), a specimen of *Dictyophorus griseus* (ENT-AM43-BO32) and one of *Neoharisea* sp. (IG32454) to cover the spectrum of the dry collections. A substantial part of the collection is composed of wet material as these specimens are best preserved in a fixation fluid solution. The specimens in these experiments were all stored within an ethanol solution and kept within this solution while pictured. Because the lens is perpendicular to the liquid’s surface, this doesn’t cause any unwanted deformation, nor reflections of the light. So specimens were never air dried prior to picturing. The specimens chosen come from the collections of Entomology and Recent Invertebrates and consist of the following specimens: a *Scolopendra galapagoensis* (IG27720), a *Solpugema hostilis* (J7-I35-MII), a *Storena formosa* (IG27675), a *Pandinus imperator* (IG32636), a *Epimeria aff georgiana* (IG32474) and a *Natantia* sp. (57810).

The specimens are chosen because it is known that they belong to a group which fluoresce when illuminated by UV light and/or because they are difficult to picture because they lost contrasting colors during the preservation process. All specimens pictured are part of the Royal Belgian Institute of Natural Sciences Collection, except for the specimen of *Natantia* sp., which is from the RMCA collection (See S1 Table for Overview).

All pictures taken are the result of focus stacking. On average 10 to 20 pictures are taken per stack. The pictures were stacked in Zerene Stacker using the PMax or DMap stacking method. The images shown are untreated unless otherwise mentioned in the results (see S2 Table for overview).

## Results

### UV 395 nm vs flash (dry)

**Fig 1:**
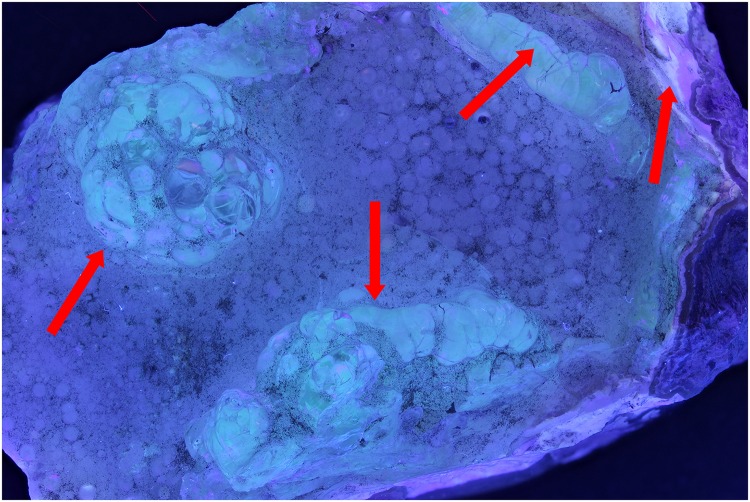
Picture of hyalite (RB 8315) with exposure of UV lights of 395 nm.

**Fig 2:**
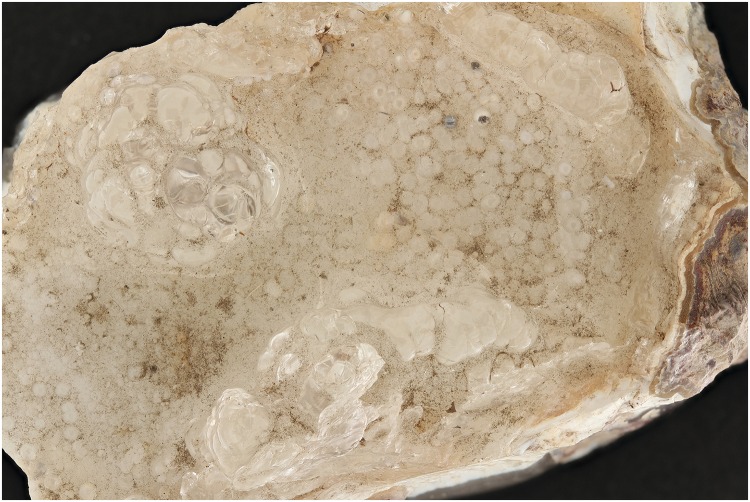
Picture of hyalite (RB 8315) with exposure of 2 flashes.

**Fig 3:**
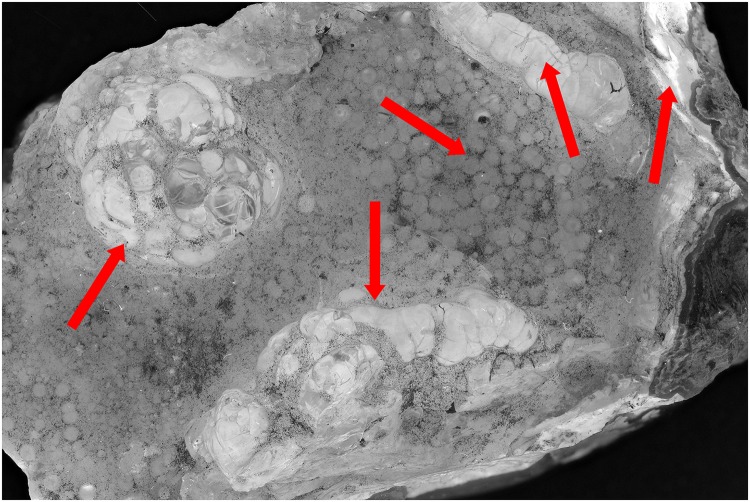
Picture of hyalite (RB 8315) with exposure of UV lights of 395 nm. The image is post processed and auto-corrected for levels and contrast and converted to grayscale.

**Fig 4:**
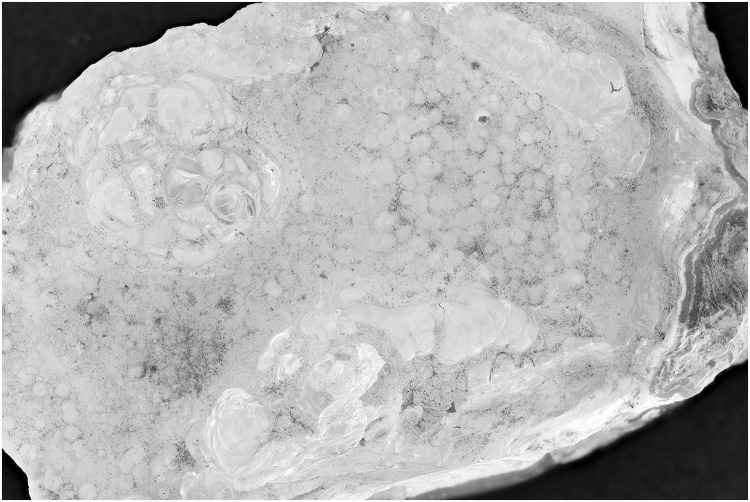
Picture of hyalite (RB 8315) with exposure of 2 flashes. The image is post processed and auto-corrected for levels and contrast and converted to grayscale.

The first test with a hyalite mineral and a UV exposure of 395 nm shows that the zones indicated by the red arrows (Fig 1) are more noticeable than the normal flash exposure in Fig 2. After the post treatment, the UV image is still better than the flash exposed one. (Figs 3 and 4). Even the zone in the middle, which was more or less equally noticeable in Figs 1 and 2, now is really contrasted in Fig 3.

### UV 365 nm vs flash (dry)

**Fig 5:**
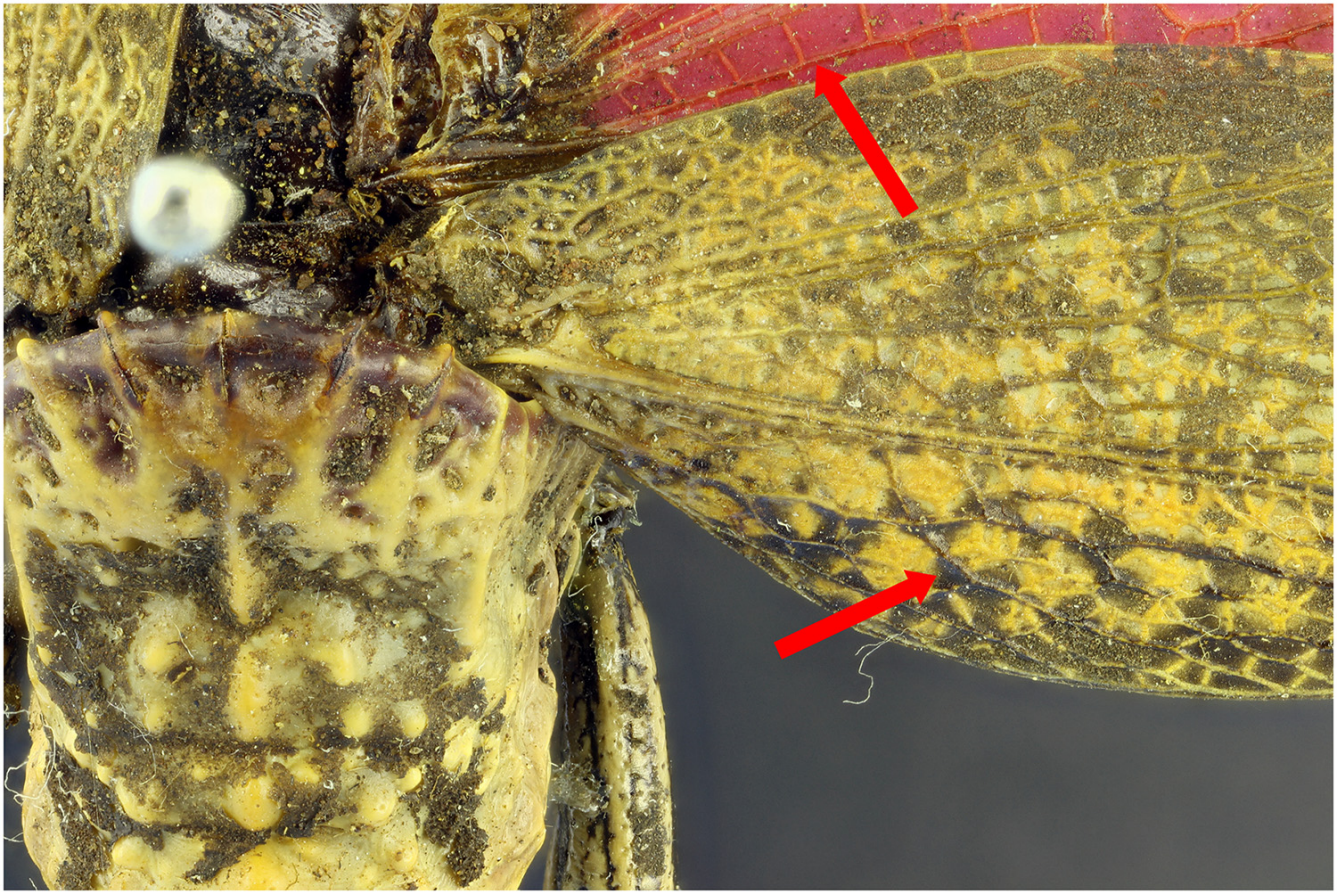
Picture of a *Dictyophorus griseus* specimen in detail with illumination of 4 UV 365 nm light bulbs. The white balance was set to custom.

**Fig 6:**
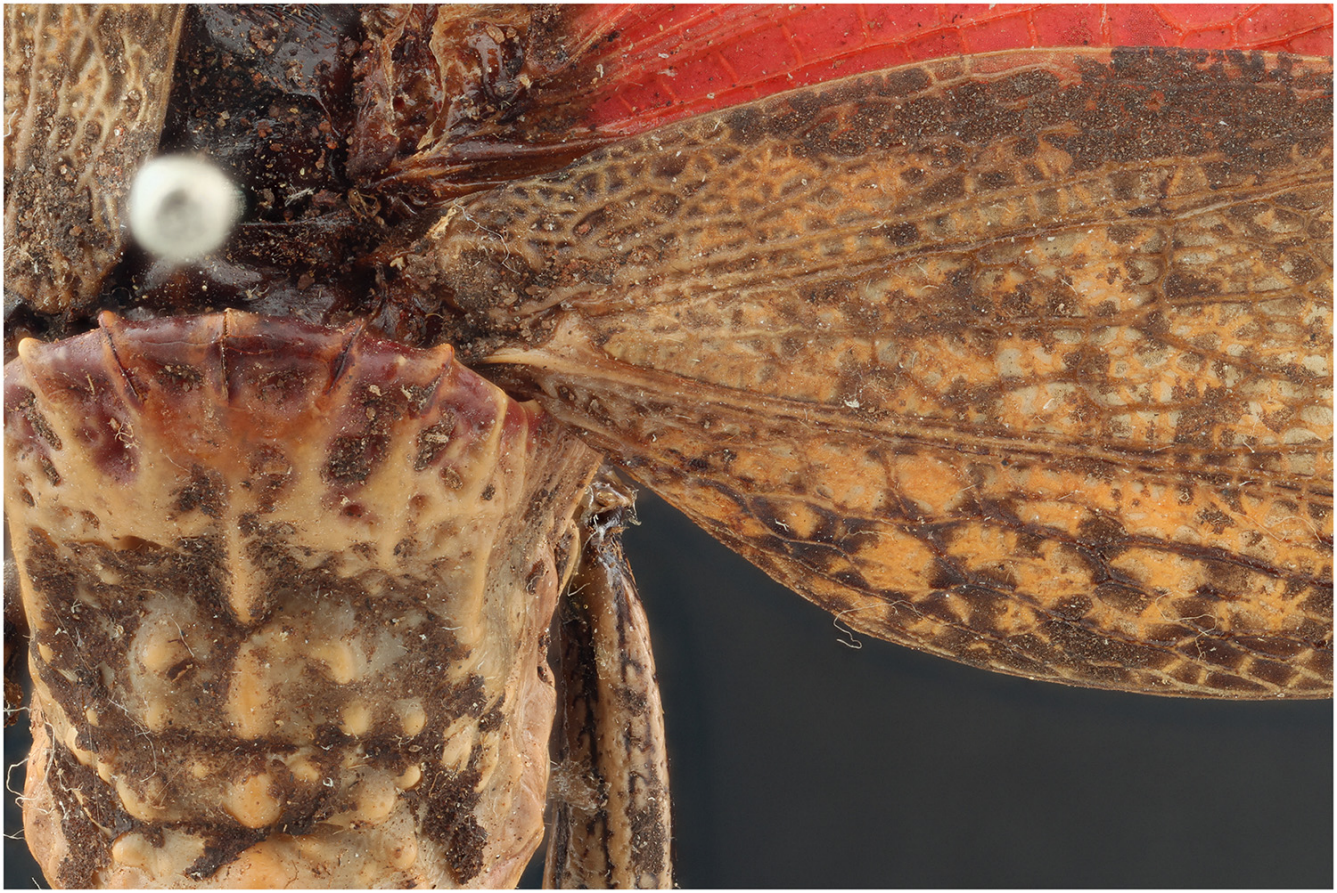
Picture of a *Dictyophorus griseus* specimen in detail with exposure of 2 flashes.

**Fig 7:**
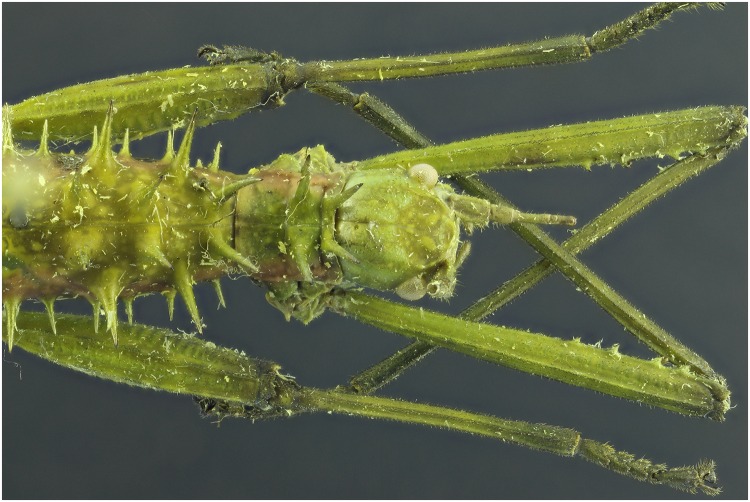
Picture of a *Neohirasea* sp. specimen with exposure of 4 UV lights of 365 nm.

**Fig 8:**
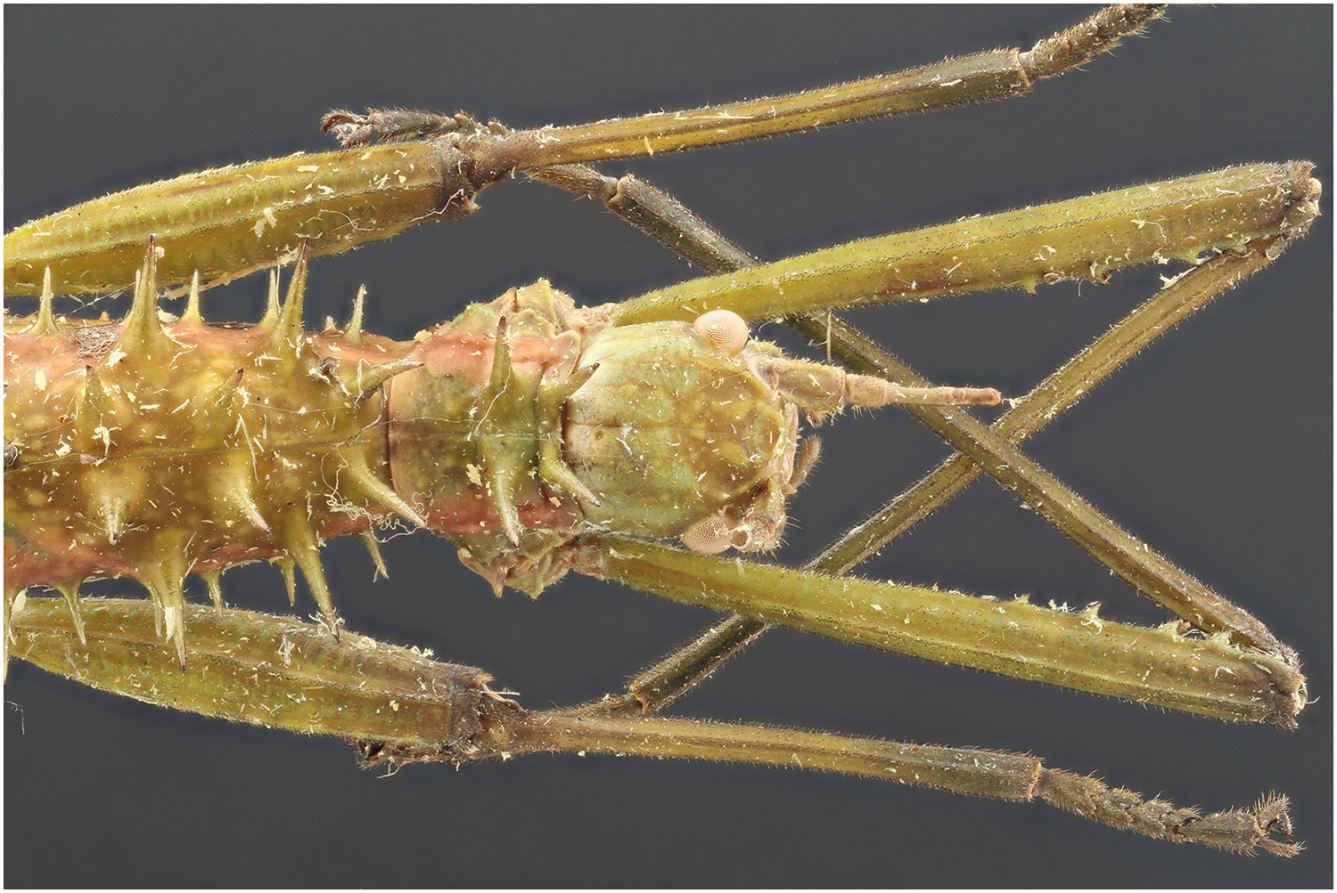
Picture of a *Neohirasea* sp. specimen with exposure of 2 flashes.

When using the 365 nm light source, specimens of the dry collection show that there is an increase in contrast and detail compared to the picture taken with flash exposure (Figs 5 and 6). In the *Dictyophorus griseus* specimen, UV light gives a more contrasted impression of the veins in the wings (see red arrows). Especially the upper part of the wing veins are difficult to distinguish in the photograph exposed with the flashes. Besides the contrast, also the color is enhanced due to UV induced fluorescence, this is very apparent in the picture comparison of the *Neoharisea* sp., where the green is enhanced in the image with the UV exposure (Figs 7 and 8).

### UV 395 nm vs flash (wet)

**Fig 9:**
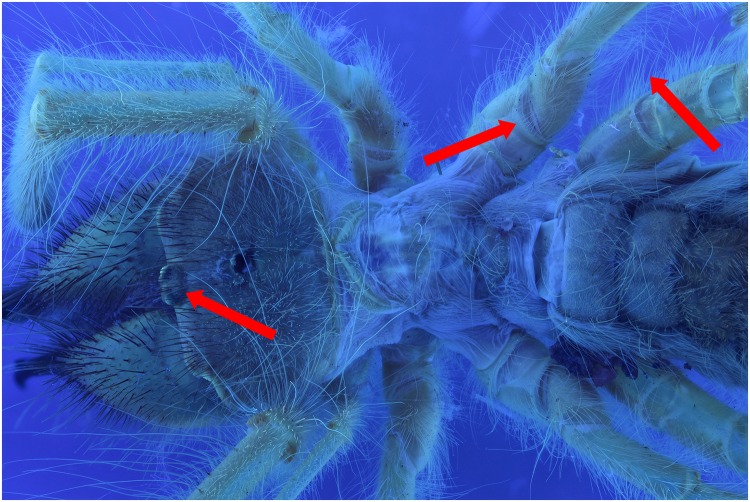
Picture of a *Solpugema hostilis* specimen with exposure of 2 UV lights of 395 nm.

**Fig 10:**
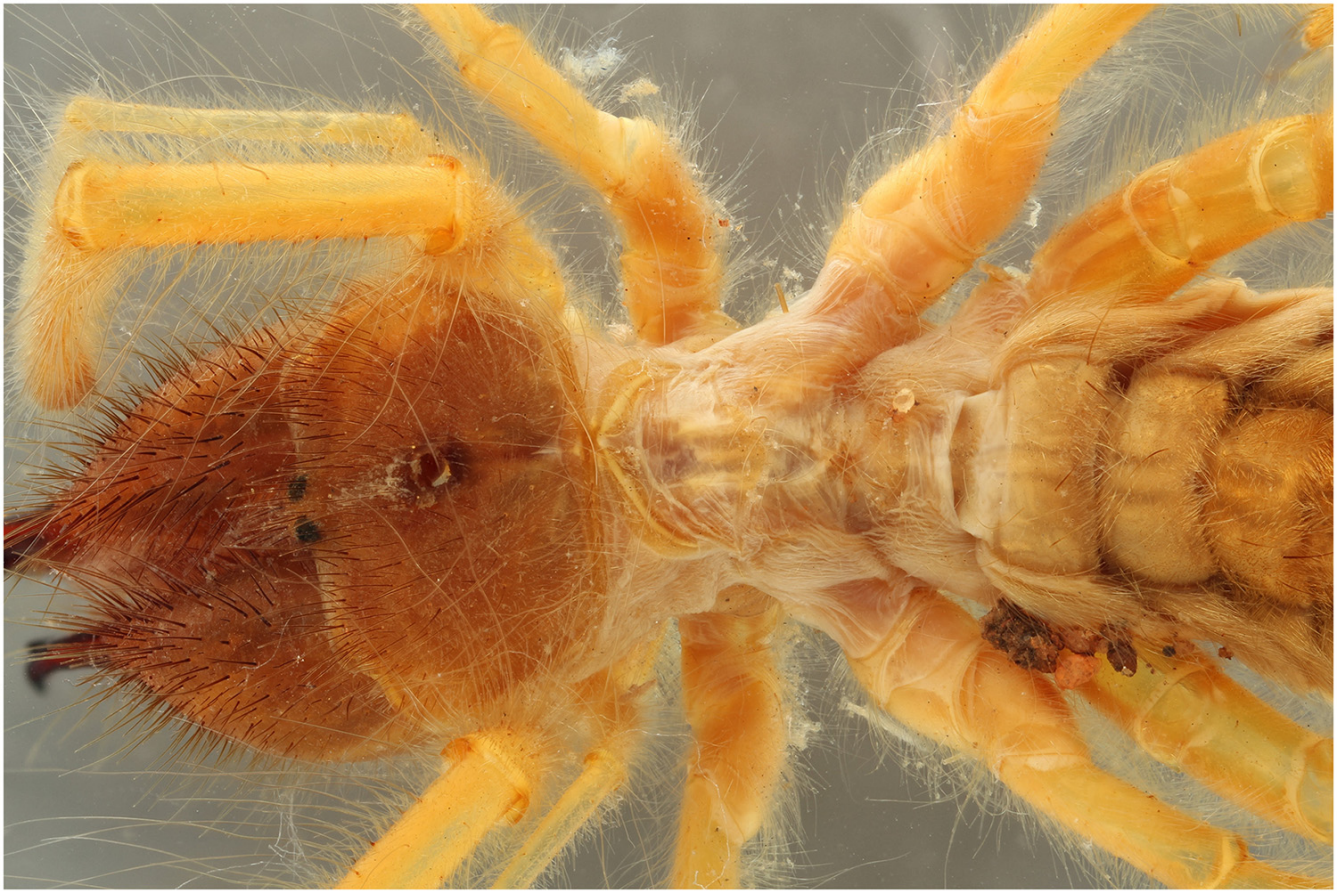
Picture of a *Solpugema hostilis* specimen with exposure of 2 flashes.

**Fig 11:**
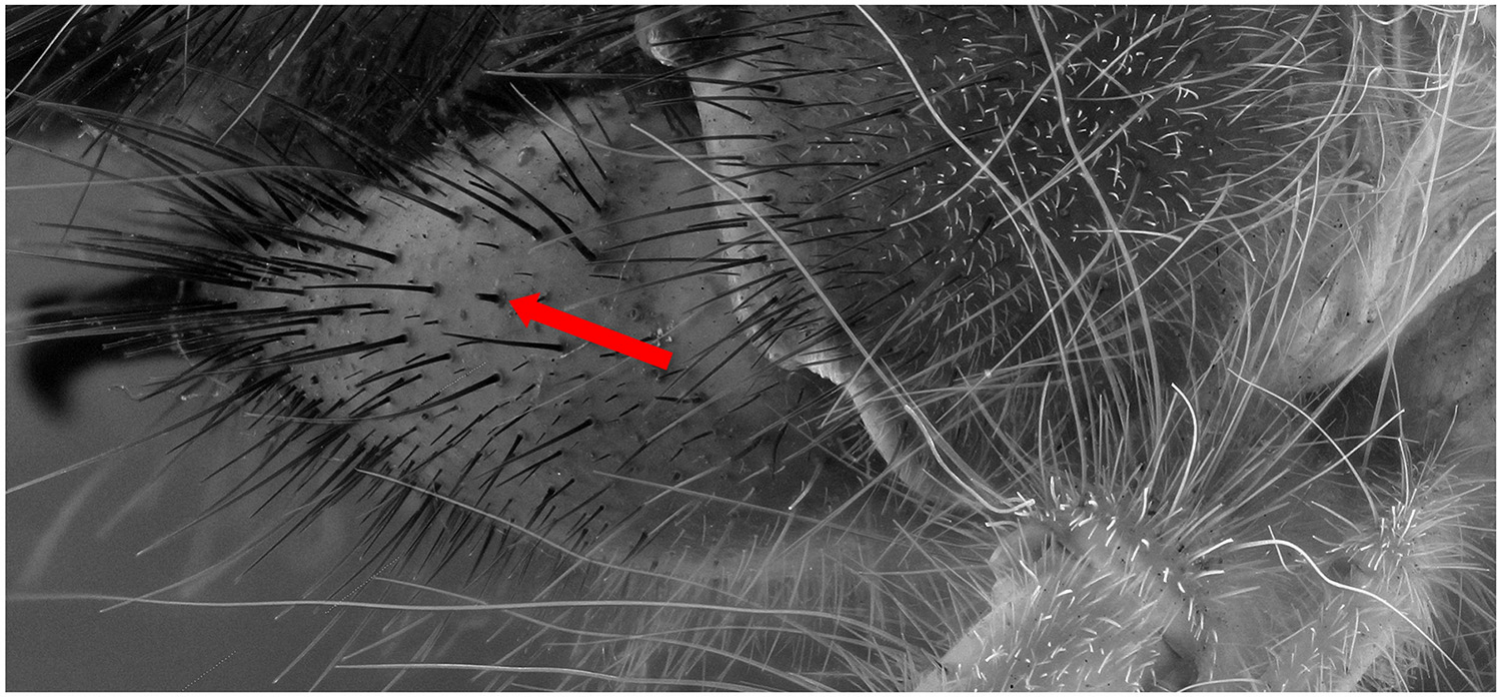
Detailed view of the Fig 9 (UV 395 nm). The picture is post treated: auto-correction for levels and contrast and grayscale conversion.

**Fig 12:**
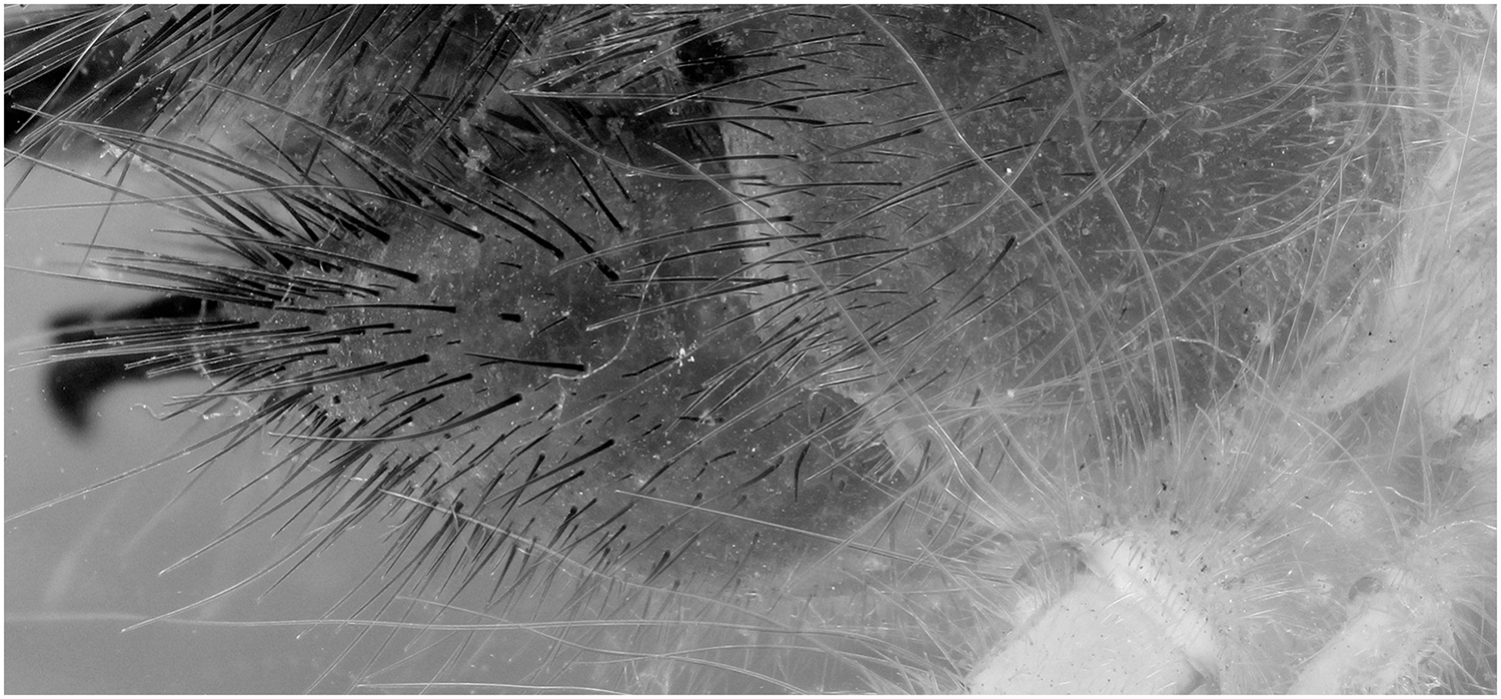
Detailed view of Fig 10 (flash). The picture is post processed using the automatic correction for levels and contrast and is converted in grayscale.

A specimen of the Solifugae, *Solpugema hostilis*, was used to test the difference in exposure between 2 UV lights of 395 nm and 2 regular flashes for a specimen preserved in an ethanol solution. Although the produced picture with the UV exposure creates a large blue tone, the differences with the regular flash exposure is large (Figs 9 and 10). All the hairs are better visible as are the different parts of the legs and the general morphology of the thorax. This is possibly an effect of the high fluorescence of the connective tissue, which creates an edge around the exoskeleton parts. Comparing an enlarged view of the two produced pictures after post-treatment, it is clear that a lot more information on the mandibles is visible in the UV picture than in the one with regular flash light (Figs 11 and 12). The picture almost gives a SEM like appearance. The UV illumination allows to see surface details like the hair bases which are not visible with the flash system as pointed out by the arrow in Fig 11.

**Fig 13:**
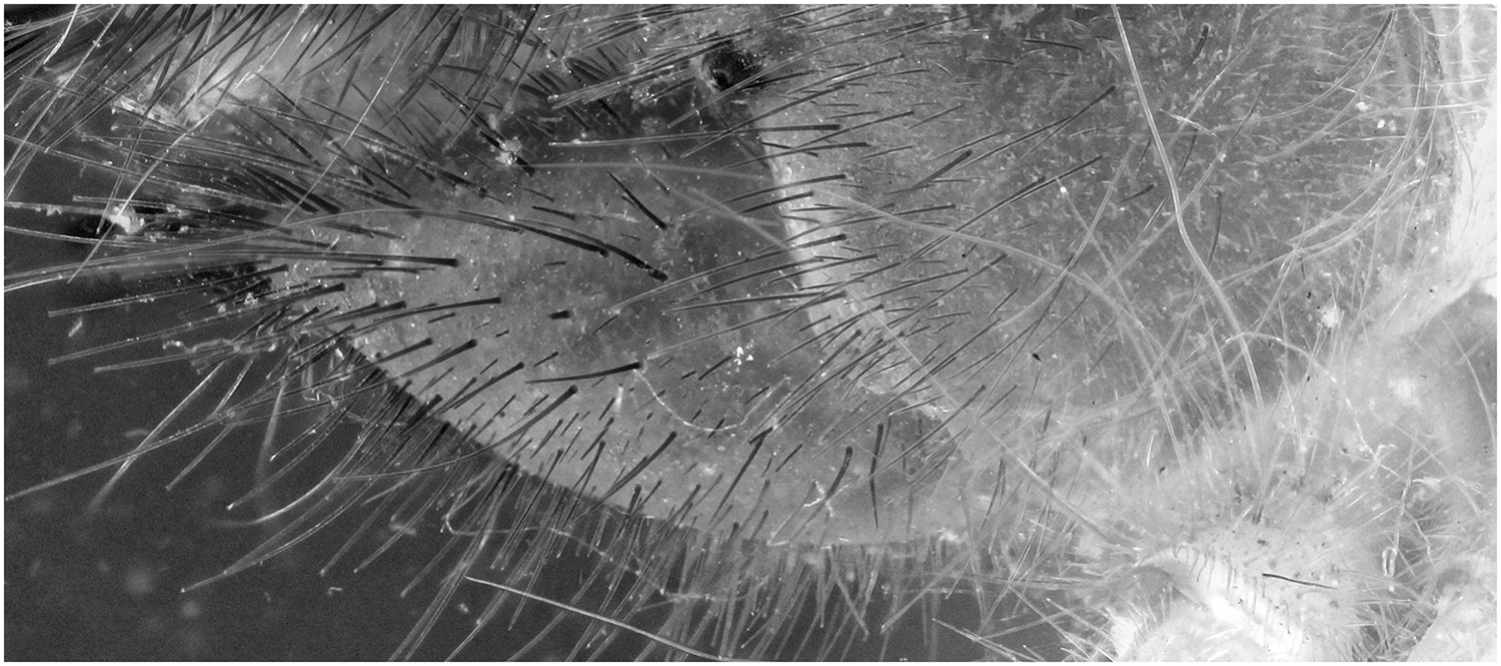
Detail of a *Solpugema hostilis* specimen. The picture is taken with continuous light exposure and post treated, converting the image into grayscale after auto correction of the levels and contrast.

The difference in detail between Figs 11 and 12 is clearly visible. To be certain, that this is due to the fluorescence produced as a reaction to the UV light source and not the difference between flash exposure and continuous lighting, a picture of the same specimen lit by LED lights was made (Fig 13). There is less detail than when pictured under UV exposure. The quality is more or less the same as with flash exposure, although not all the hairs on the mandibles are as sharp.

### UV 365 nm vs flash (wet)

**Fig 14:**
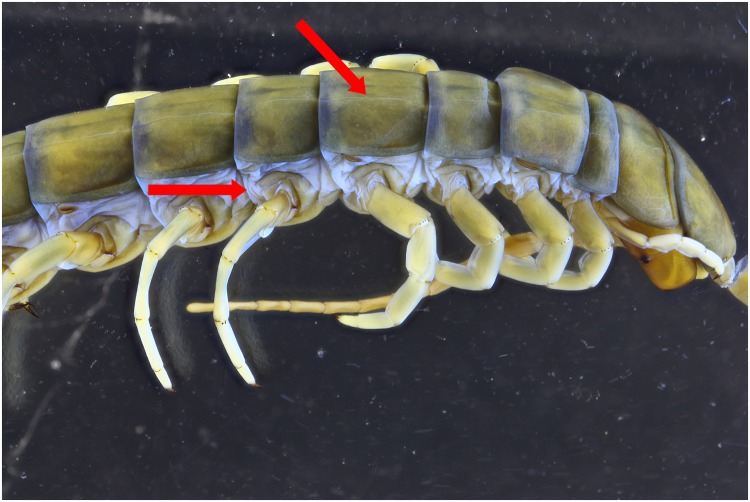
Picture of a *Scolopendra galapagoensis* specimen. The specimen is illuminated by 4 light bulbs UV 365 nm and custom white balance was used.

**Fig 15:**
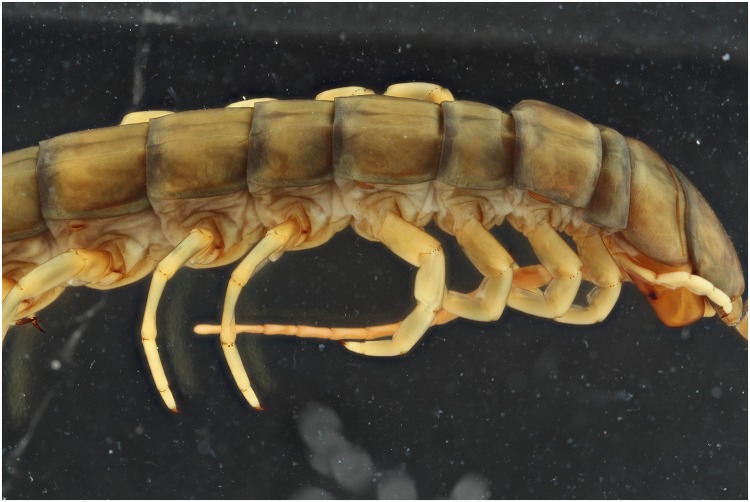
Picture of a *Scolopendra galapagoensis* specimen with exposure of 2 flashes.

**Fig 16:**
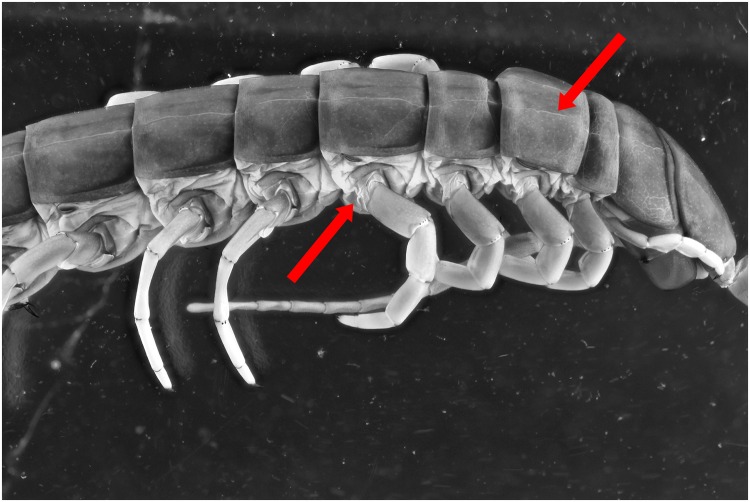
Picture of a *Scolopendra galapagoensis* specimen with exposure of 4 UV light bulbs of 365 nm. Custom white balance was used. The picture is post processed using the auto levels function and converted into grayscale.

**Fig 17:**
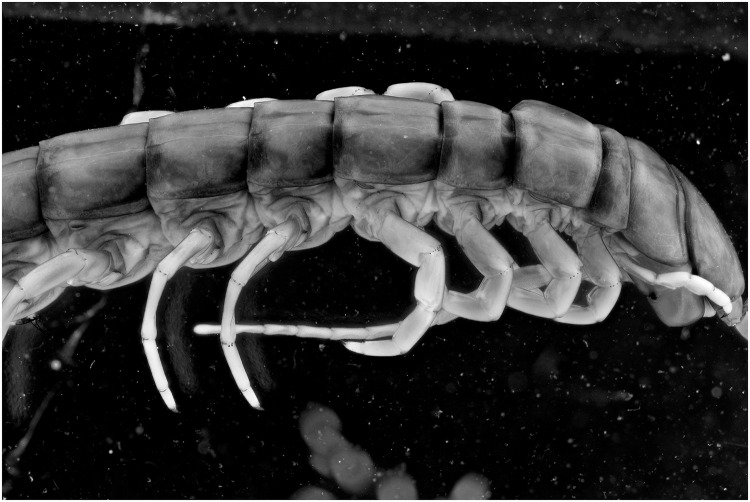
Picture of a *Scolopendra galapagoensis* specimen with exposure of 2 flashes. The picture is post processed using the auto levels function and converted into grayscale.

To test the effect of the 365 nm light setup for specimens within ethanol as well several pictures are taken. There is a gain in contrast noticeable on the UV picture (Figs 14 and 15). The red arrows indicate areas with more details, as small micro cracks on the tergites and folds near the legs. Here as well, the pictures are post treated to analyse if the gained detail isn’t the effect of difference in color. The treated pictures show that there still is a noticeable difference between the two light sources (Figs 16 and 17).

### UV 365 nm vs UV 395 nm

**Fig 18:**
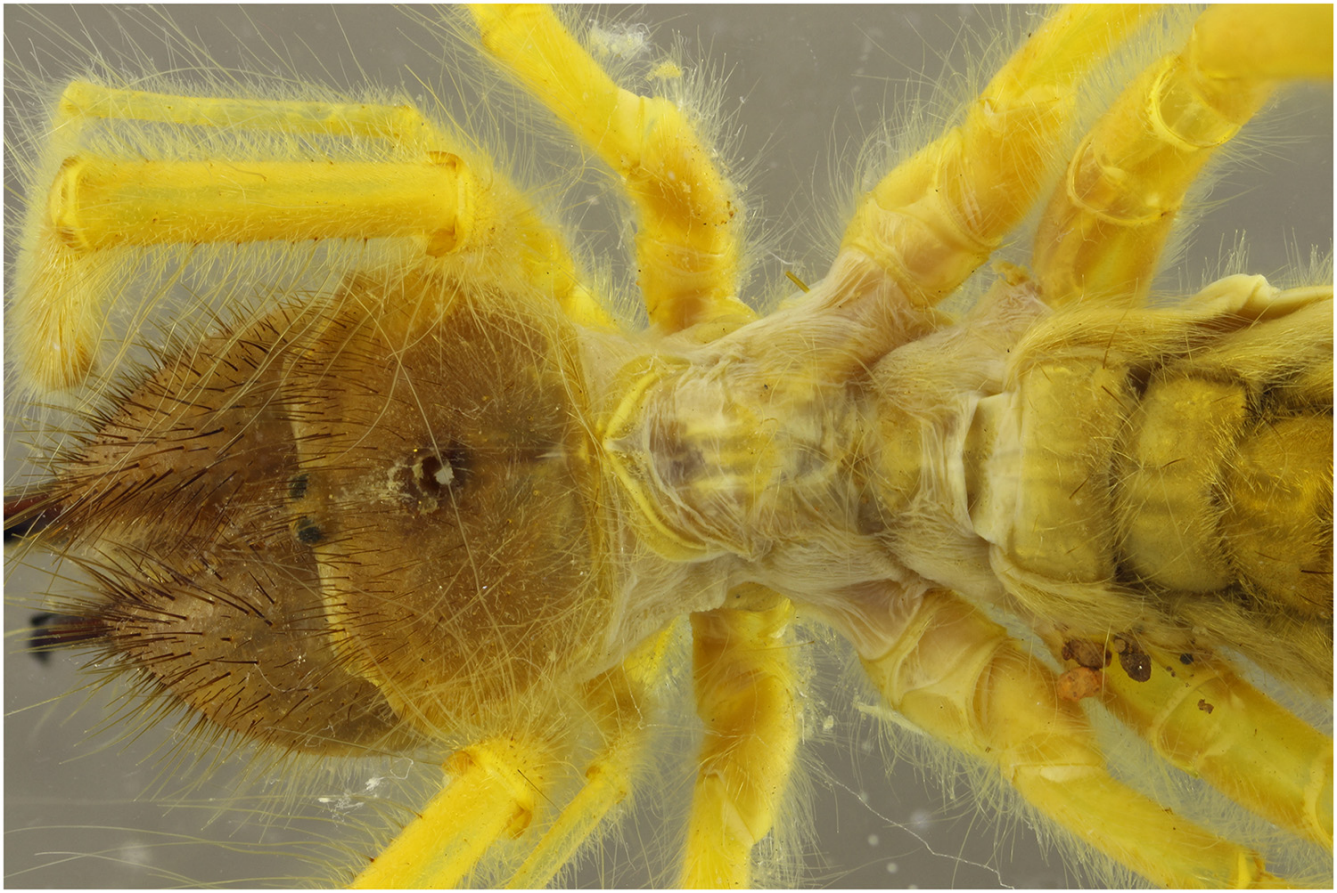
Picture of a *Solpugema hostilis* specimen with an exposure of 4 UV lights of 365 nm.

**Fig 19:**
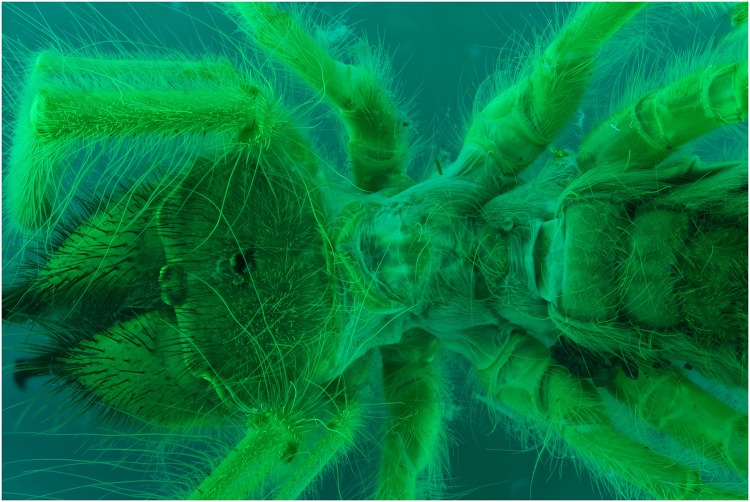
Picture of a *Solpugema hostilis* specimen with an exposure of UV lights of 395 nm.

The 365 nm picture creates a less blue look due to being a more bright white light, while the black light is very blue, when using the auto white balance (Figs 18 and 19). Besides the difference in color of the light source, there is a large difference in exposure. Using the 365 nm lights, exposure time decreases by a factor 10, which speeds up the process in combination with focus stacking quite drastically.

The picture made using the 395 nm (Fig 19) delivers a bit more detail of the hairs than when using the 365 nm (Fig 18). It looks like the hairs react more to 395 nm, while the exoskeleton itself shows fluorescence when exposed to light of 365 nm.

### UV 365 nm 4 bulbs vs 2 bulbs

**Fig 20:**
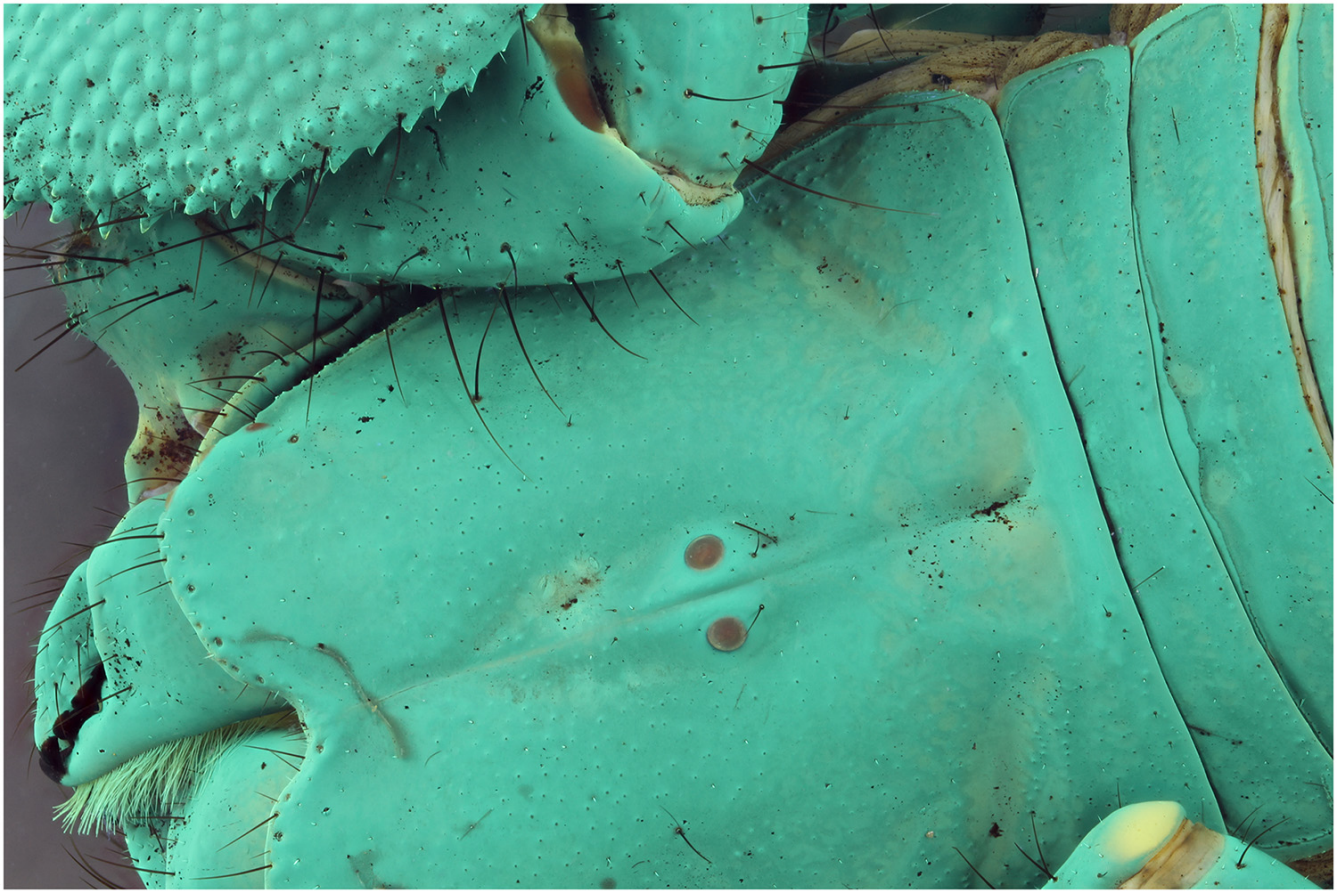
Picture of a *Pandinus imperator* specimen with exposure of 4 light bulbs of 365 nm.

**Fig 21:**
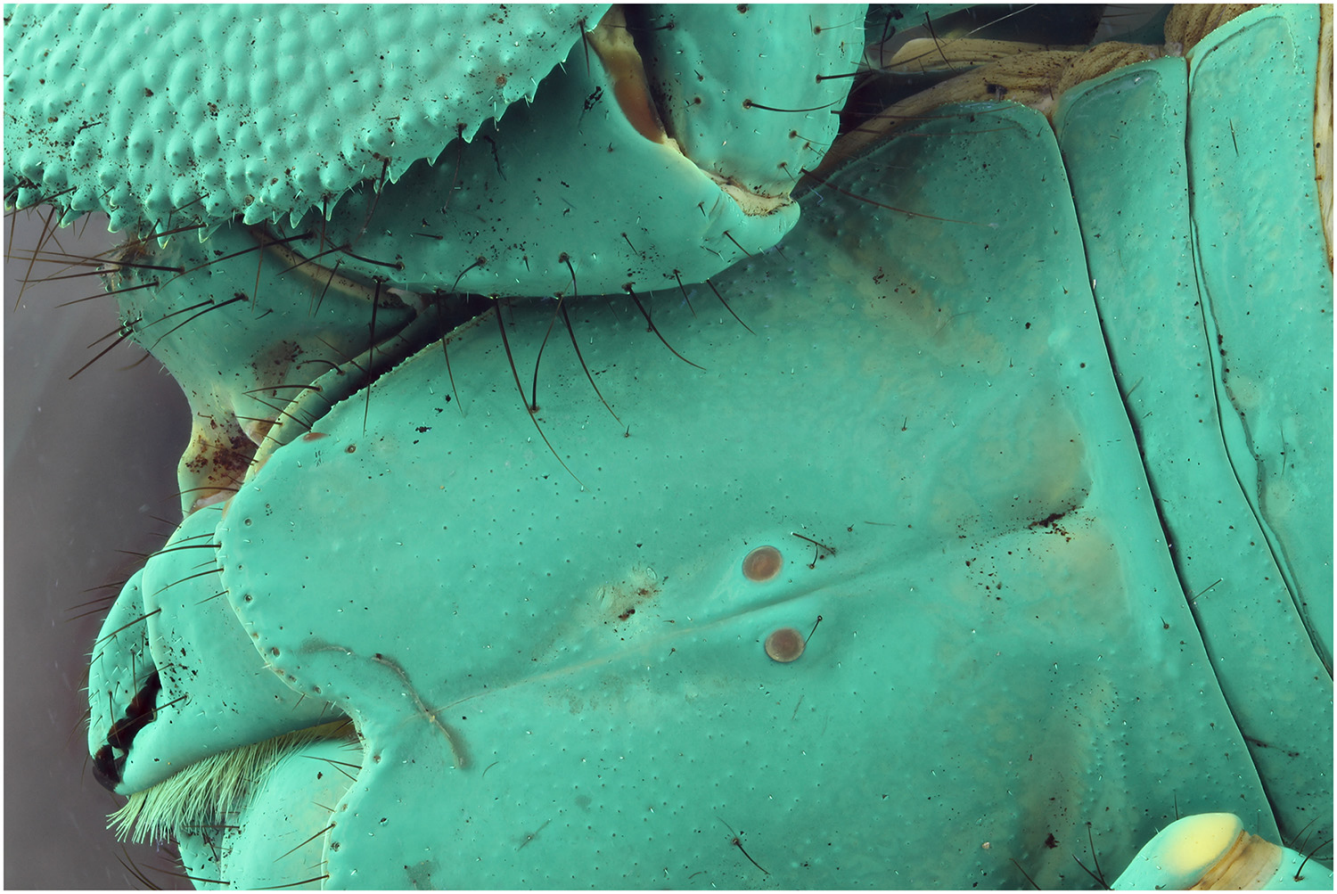
Picture of a *Pandinus imperator* specimen with exposure of 2 light bulbs of 365 nm.

**Fig 22:**
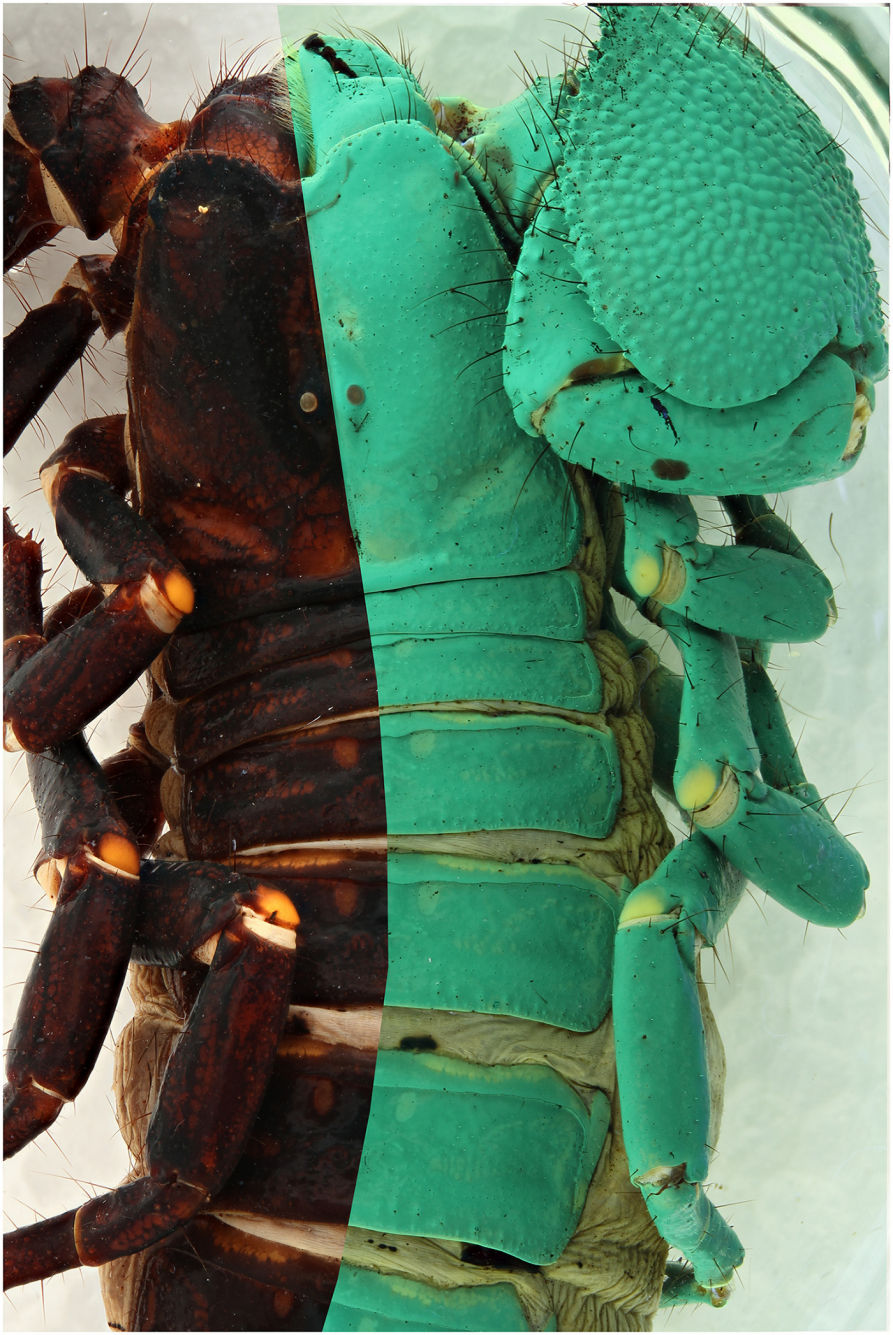
A composite picture of *Pandinus imperator*. It is exposed by flash on the left side of the picture and by 2 UV 365 nm at the right side.

*To check whether the* general setup of the 365 nm UV nail dryer, which comes with 4 light bulbs, generates sufficient ambient light, when using only 2 of the bulbs, a test using a specimen of the wet collection is performed. For this test, the exposure time doubles as expected when using 2 lamps instead of 4, but still is quite manageable as the exposure for 2 lamps is only 0.5s (Figs 20 and 21). More importantly, the level of detail is the same and still hugely different from the regular flash exposure as the composite picture (Fig 22) shows.

### UV + flash combination

**Fig 23:**
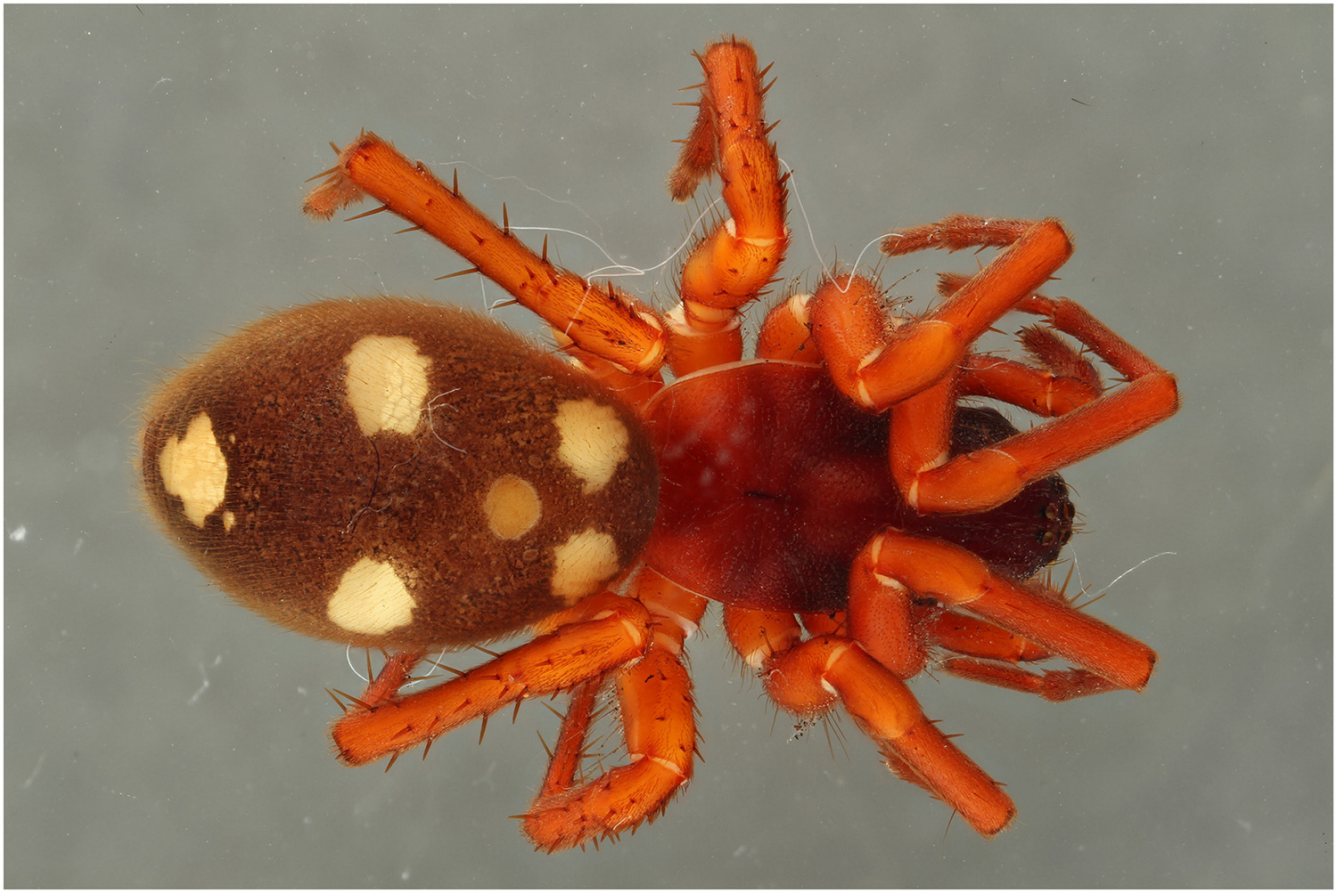
Picture of a *Storena formosa* specimen with exposure of 2 flashes.

**Fig 24:**
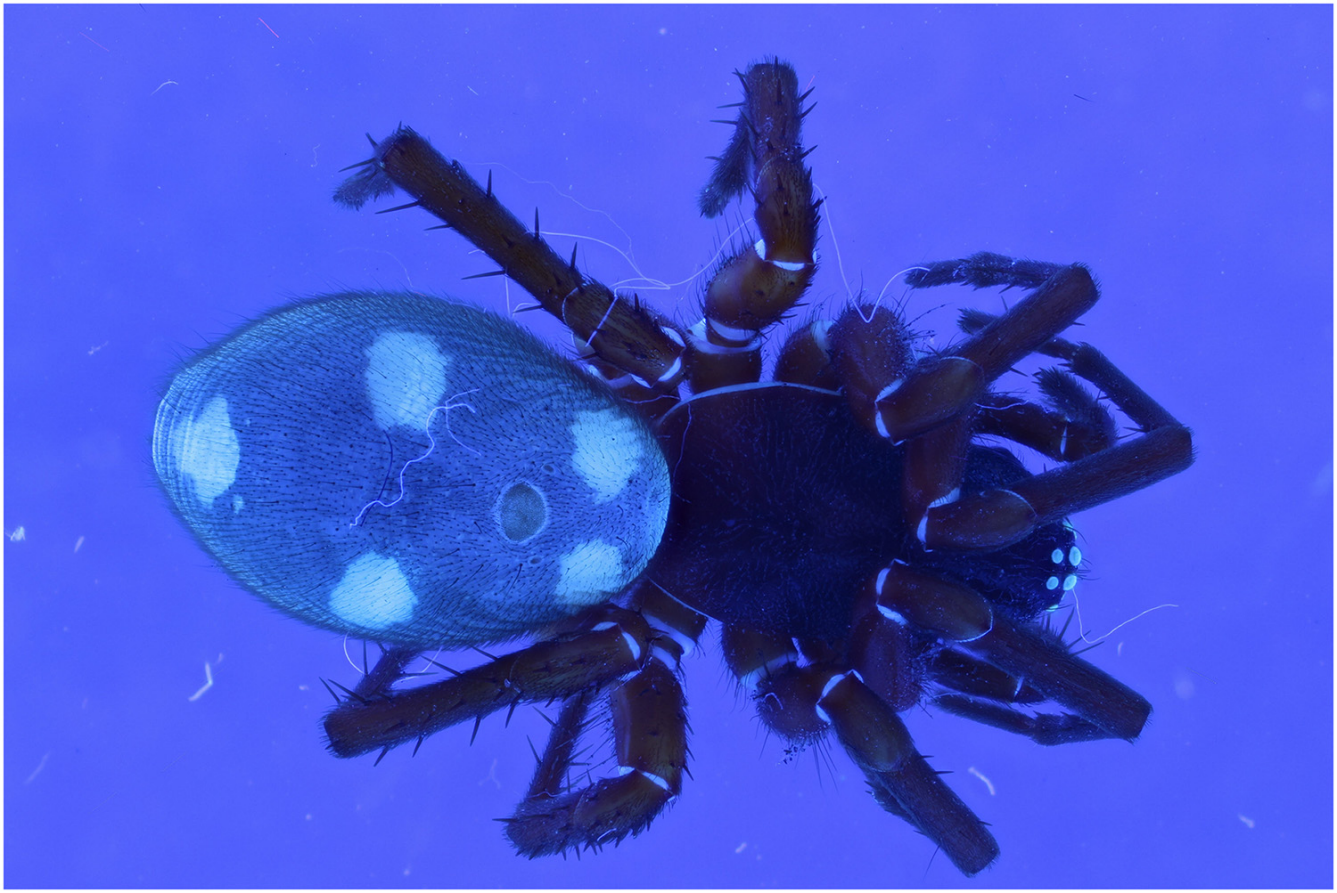
Picture of a *Storena formosa* specimen with exposure of 2 UV lights of 395 nm.

**Fig 25:**
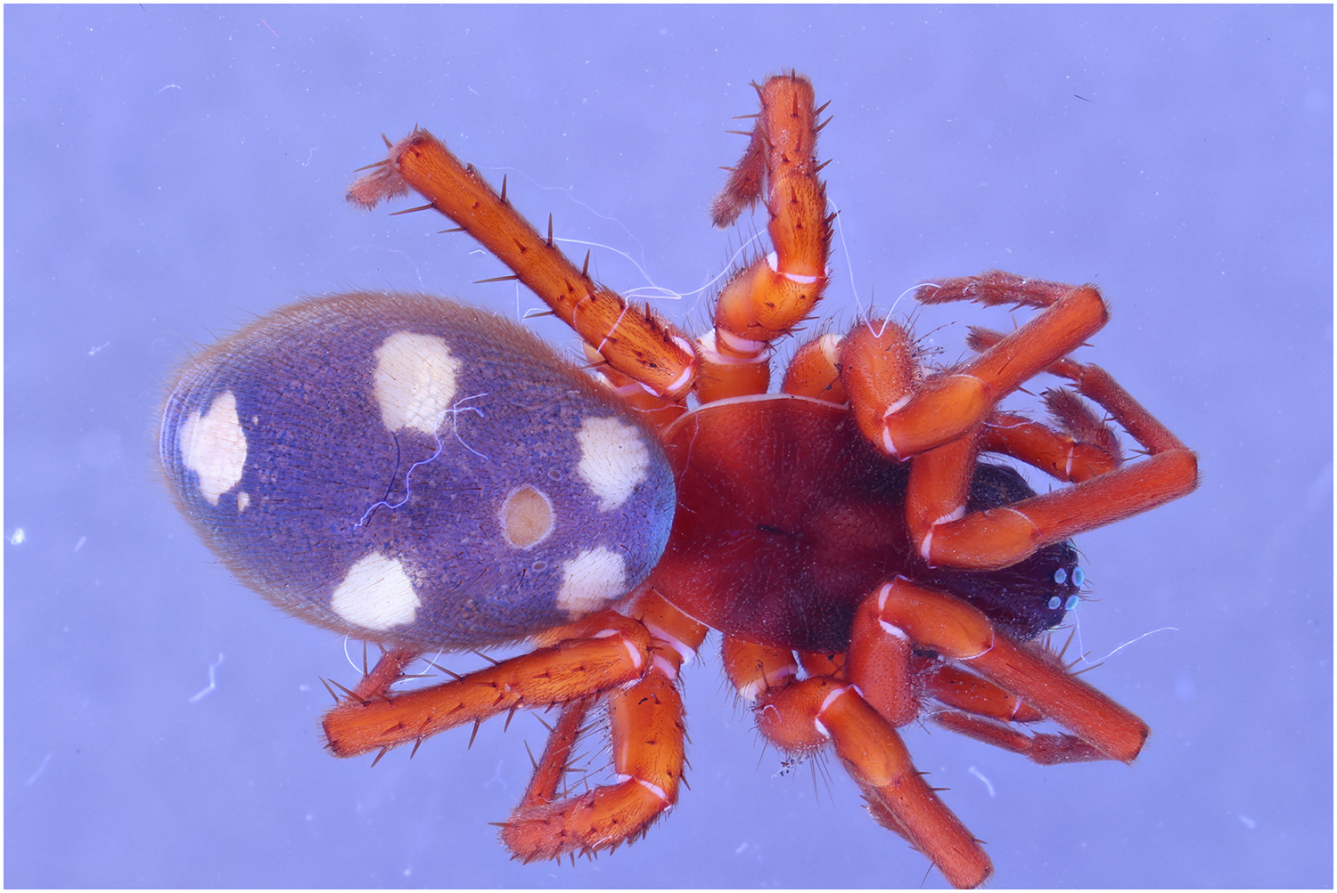
Picture of a *Storena formosa* specimen with exposure of both 2 flashes and 2 UV lights of 395 nm.

**Fig 26:**
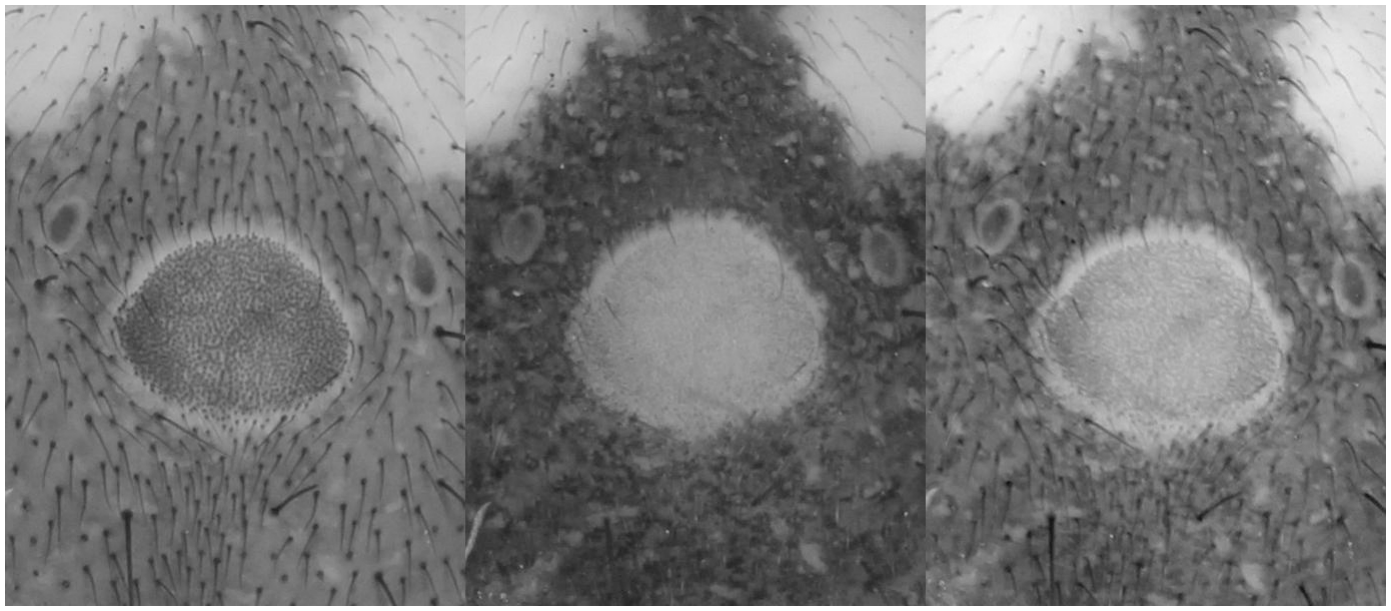
Detailed view of the abdomen of Storena formosa. From left to right: UV 395 nm, Flash, Combination of UV 395 nm + Flash. All the detailed views are retrieved from figs 23 to 25 after post treatment using the auto levels, auto contrast and converted into grayscale.

Some specimens do not show a lot of UV induced fluorescence. A test was performed to check whether using both flash and UV illumination in a single capture could solve the issue of having underexposed or less detailed parts of the specimen in the final image (Figs 23, 24 and 25). In this test the 395 nm light source was used. The exposure was a little bit shorter during the combination shot, 6s instead of 10s, than when using only the UV lights. The main benefit is that with the 395 nm the areas which normally do not lit up (Fig 24) are normally exposed now. Even though there is a gain of detail visible on the abdomen (Fig 26), it isn’t as detailed as the UV picture, at least in this specimen.

### UV exposure with and without yellow filter

**Fig 27:**
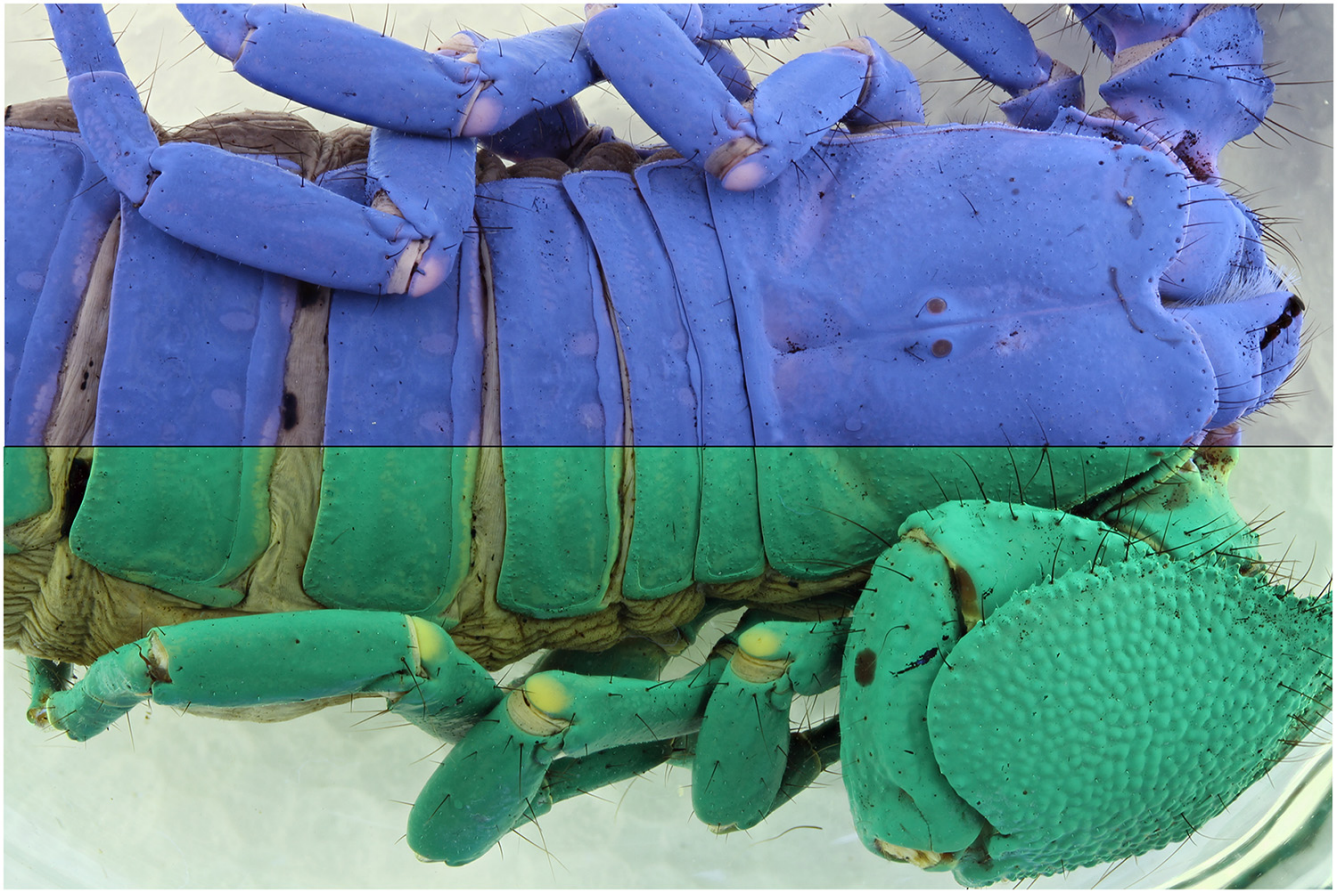
Picture of a *Pandinus imperator* specimen with exposure of 4 UV lights of 365 nm. A stack of pictures is taken with a yellow filter (top) and without one (bottom).

**Fig 28:**
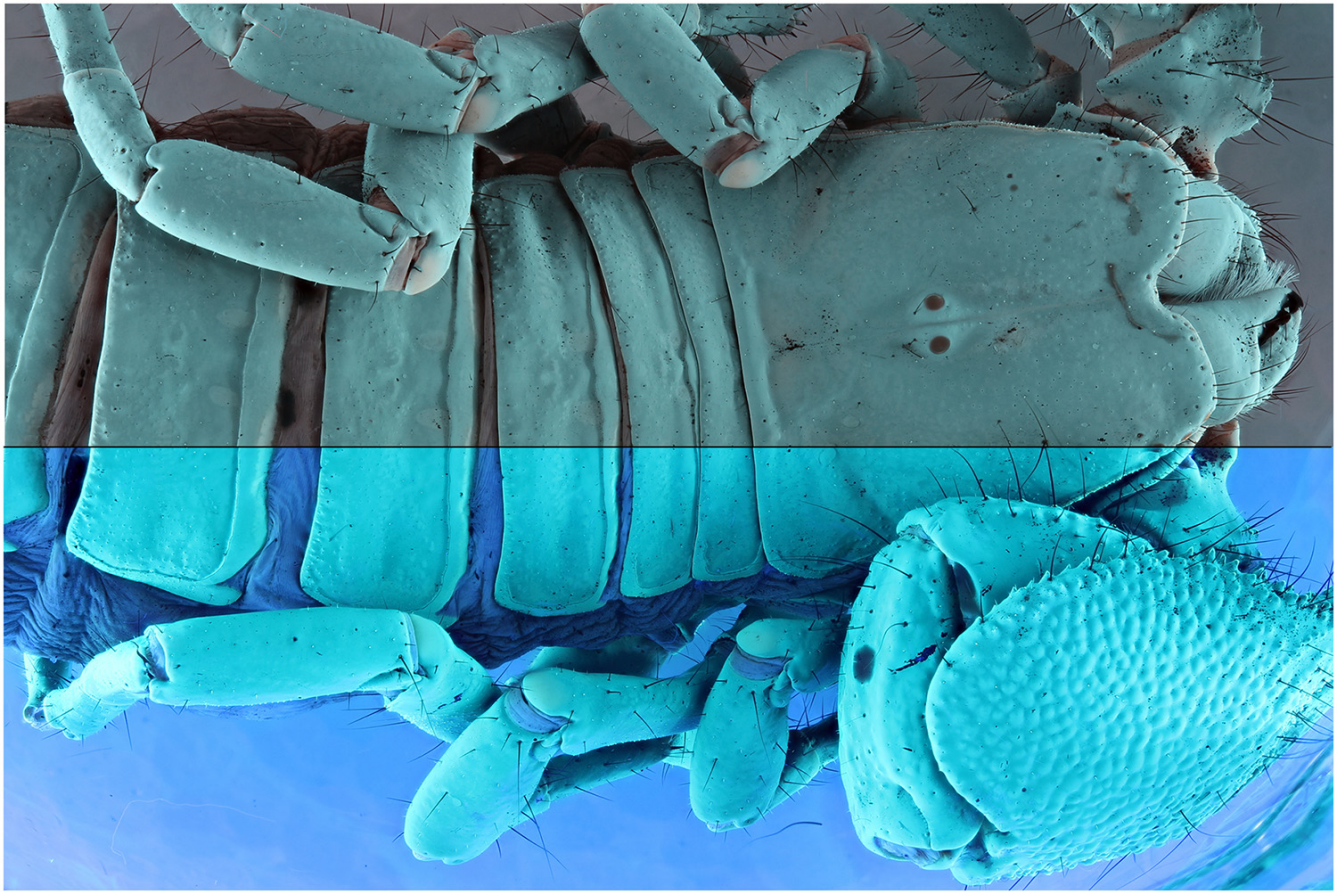
Picture of a *Pandinus imperator* specimen with exposure of 2 UV lights of 395 nm. A stack of pictures is taken with a yellow filter (top) and without one (bottom).

Using a yellow filter helps to block the blue light which is very apparent with the UV black light, tests using such a filter should reveal how this might affect the final pictures. When using the 365 nm light source, not a lot changes besides the different color of the specimen (Fig 27). The yellow filter in combination with the 395 nm lights, removes all the blue from the background and gives the specimen a stone washed appearance (Fig 28). It also creates more contrast and a background as if it is pictured using regular light, especially in the pictures taken with the 395 nm UV bulbs.

### UV vs flash in low contrast specimens

**Fig 29:**
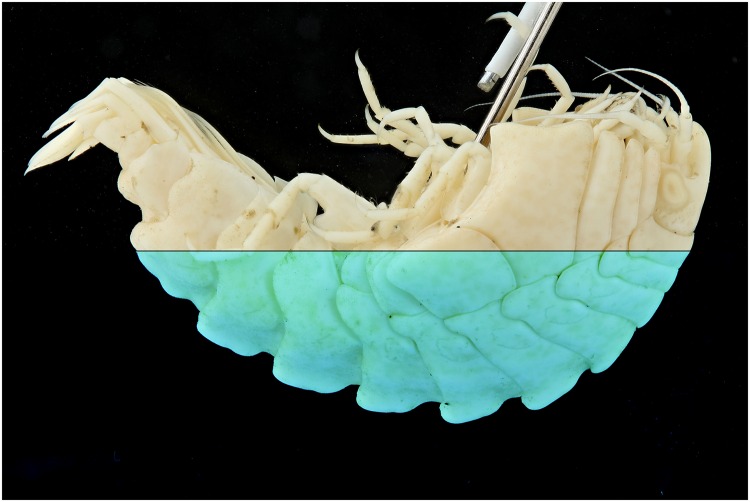
Combined picture of an *Epimeria aff*. *georgiana* specimen. The flash lights exposed picture above and the UV (365 nm) exposure is shown below.

**Fig 30:**
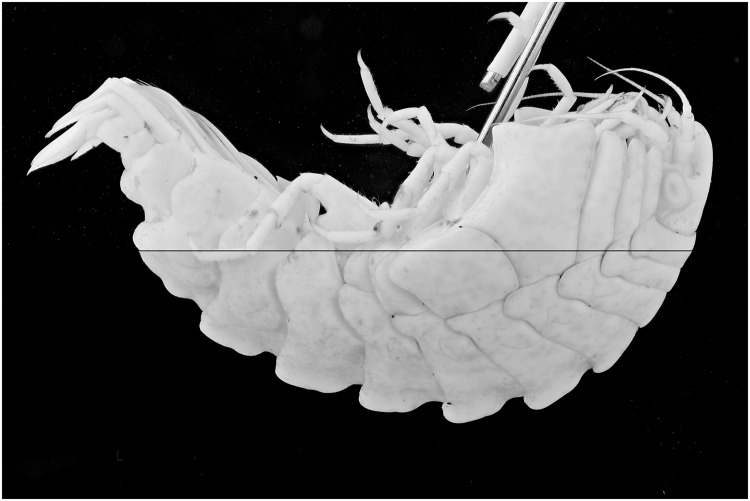
Combined picture of an *Epimeria aff*. *georgiana* specimen. The flash lights exposed picture above and the UV (365 nm) exposure is shown below. The pictures are corrected for the levels and desaturated using the average option in GIMP.

**Fig 31:**
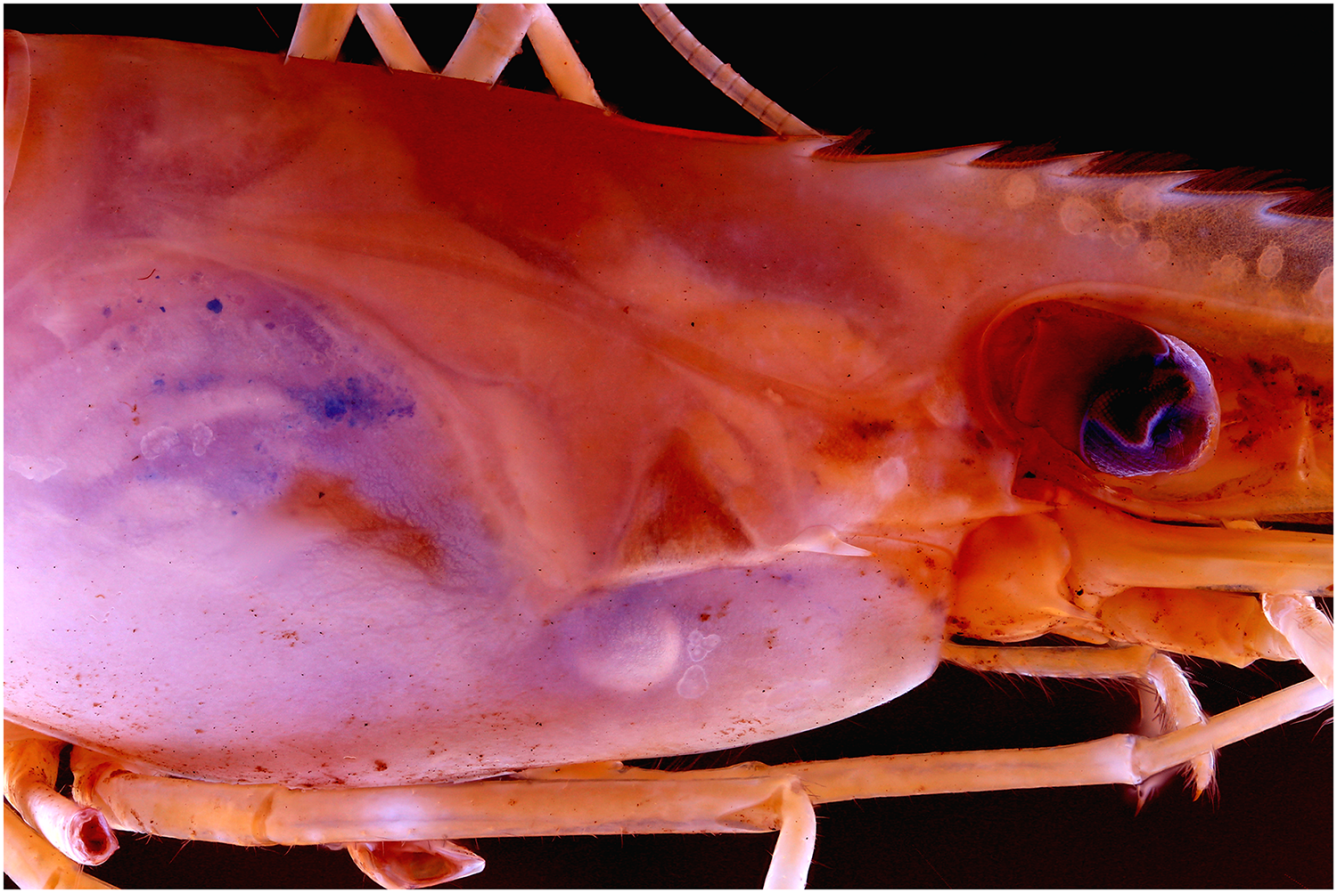
Picture of a *Natantia* sp. with custom white balance and auto levels at 395 nm.

**Fig 32:**
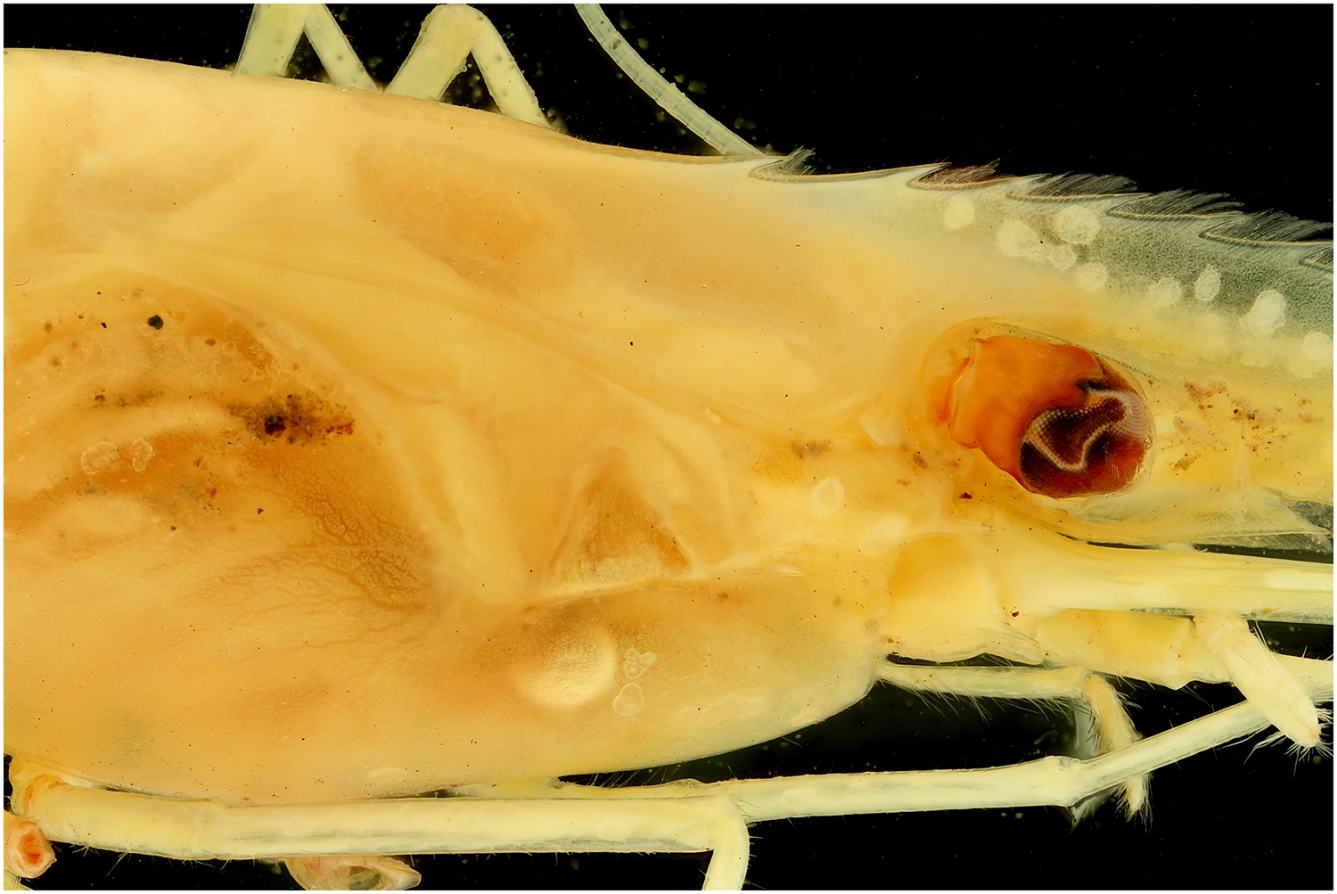
Picture of a *Natantia* sp. using auto levels and flash illumination.

Specimens which lack colors, for instance due to the preservation process, are difficult to picture. In this test a *Natantia* sp. and an *Epimeria aff*. *georgiana* specimen, both uniformly colored after preservation, are illuminated by the 395 nm and 365 nm respectively to examine whether UV exposure might add contrast to the final pictures At 395 nm, the *Epimeria aff*. *georgiana* specimen didn’t show any fluorescence. But there was a faint fluorescent reaction when using the 365 nm (Fig 29). This eventually improved the contrast and revealed a little bit more pattern information as seen on Fig 30 after post-treatment. However, the *Natantia* sp., showed several zones of fluorescence, which were really revealed using the custom white balance function (Figs 31 and 32). Further tests might be used to show possible differences within cryptic species or within complexes with cryptic species.

### De-filtered camera

**Fig 33:**
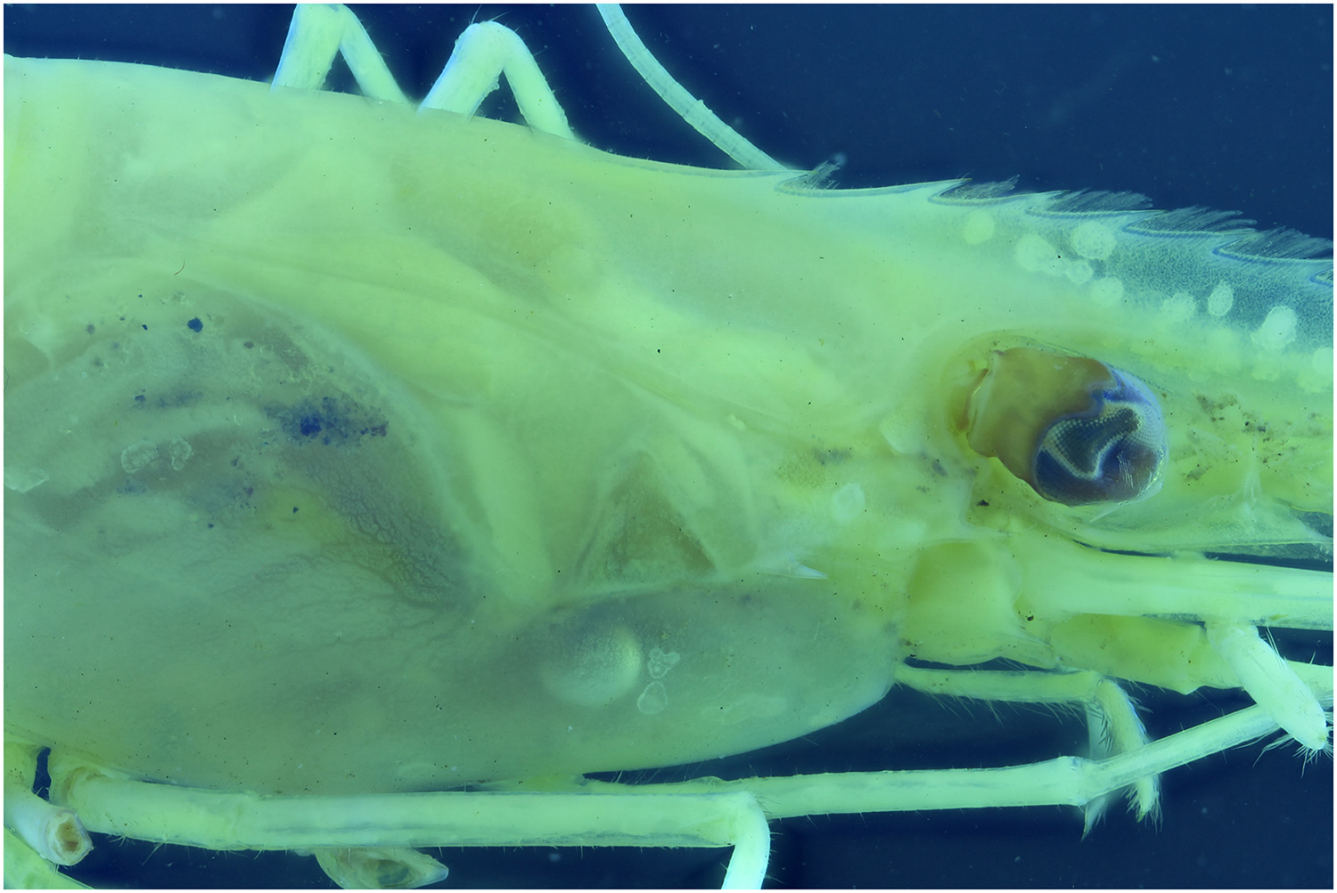
Picture of a *Natantia* sp. specimen, using a default Canon 700D with 365 nm exposure.

**Fig 34:**
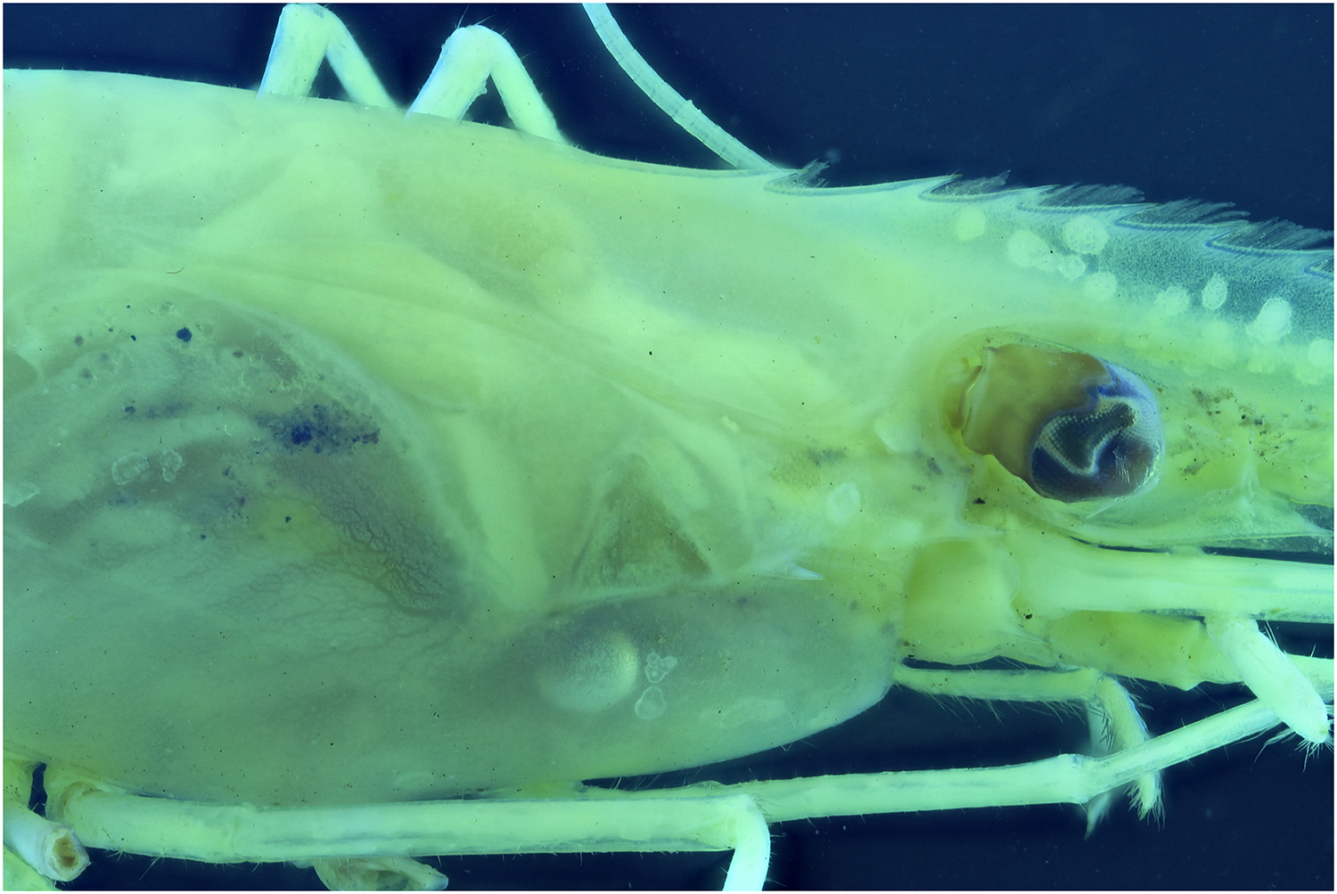
Picture of a *Natantia* sp. specimen, using a de-filtered Canon 600D with 365 nm exposure.

**Fig 35:**
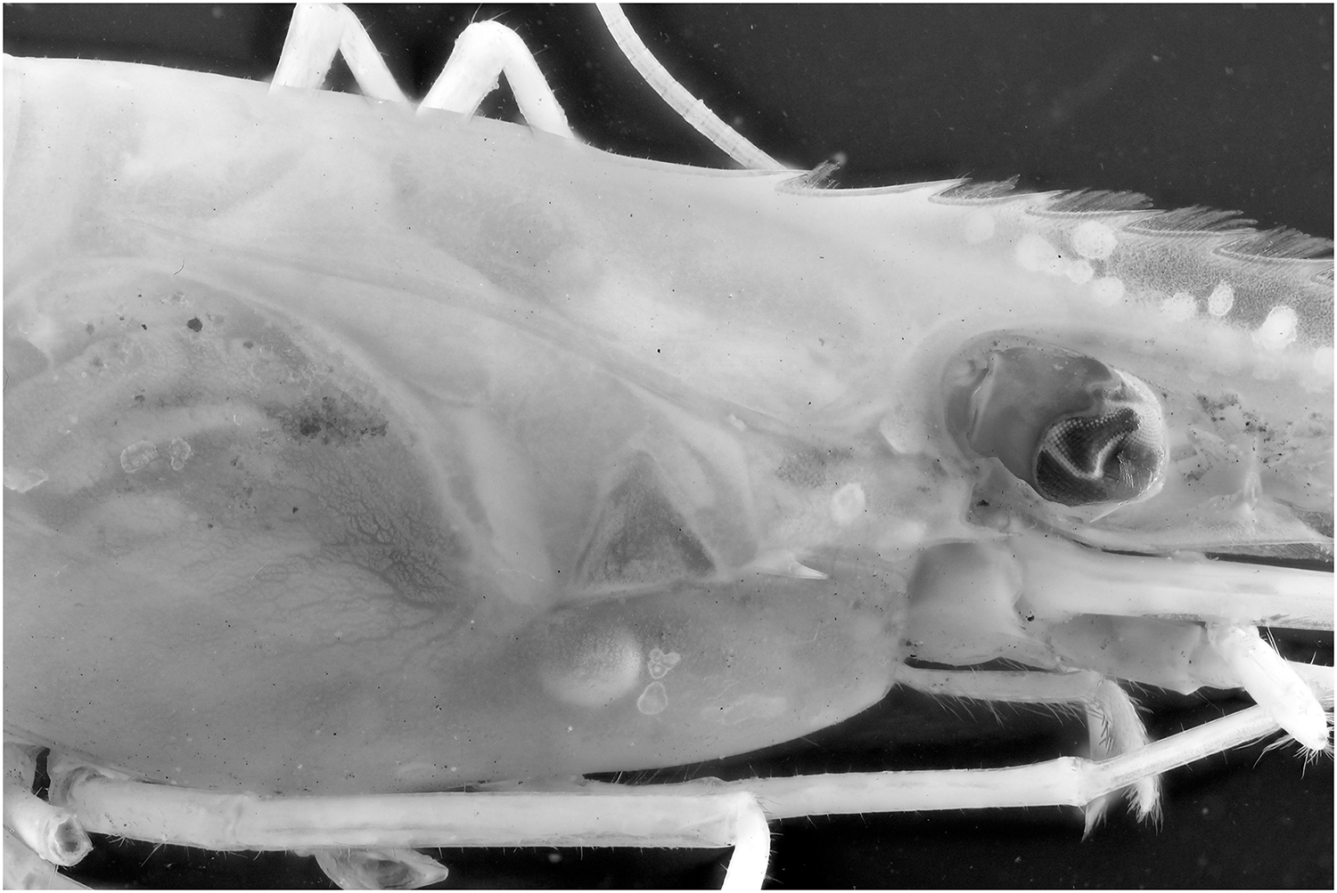
Picture of a *Natantia* sp. specimen, using a default Canon 700D with 365 nm exposure. The image is post treated for auto levels, contrast and grayscale conversion.

**Fig 36:**
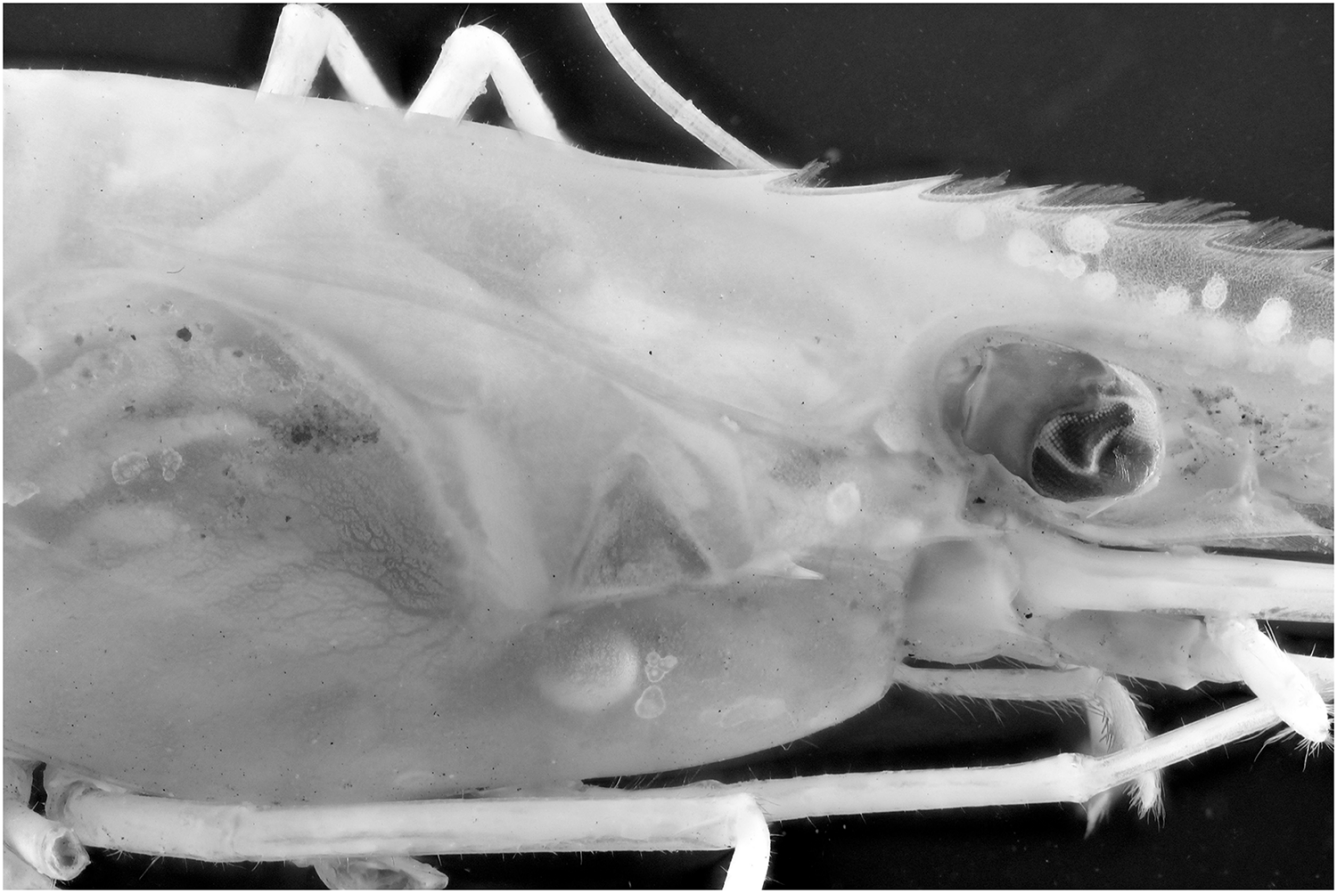
Picture of a *Natantia* sp. Specimen, using a de-filtered Canon 600D with 365 nm exposure. The image is post treated for auto levels, contrast and grayscale conversion.

To see what the effect on a picture is with UV exposure, a de-filtered Canon EOS 600D was used. Looking at the pictures (Figs 33, 34, 35 and 36), there isn’t any difference noticeable. At the same settings, the pictures of the de-filtered camera looks a little bit brighter, which does makes sense as the internal UV filter is removed, but for the rest any differences are visible.

## Discussion

Fluorescence is a well known reaction in many animals (Spiders: [9], [10]; Insects: [11], [12]; Crustaceans: [13]; Birds: [2], [14]; Fish: [15]; Fossils: [16], [17]; Plants: [18], [19], [20]; etc.) and materials, which are well represented in Natural History museums. The question remained of course, whether fluorescence still occurs after years of storage. The tests performed proved that it still does even when specimens are preserved within an ethanol solution. It is also quite clear that when a fluorescent reaction to UV occurs, it provides more detail for the parts that lit up compared to traditional flash photography. The pictures taken with LED exposure, made clear that the gain in detail seen in the pictures with UV exposure is the effect of the fluorescence of the specimen and not because it is a continuous light source. The examples show that both specimens stored in ethanol or preserved in a dry form do react to the UV light. This offers great perspective for digitization programs to investigate new ways of preserving as much information as possible of a collection specimen. If a decision has to be made which of the tested UV light sources proves out to give the best results, it is a tough call. The 365 nm lights emit a more natural light, which results in a more realistic picture. Because the light emitted from the lamps used is brighter, the shutter speed is significantly faster. This hugely reduces the entire time of taking stacks of pictures up to a factor ten. However, for some specimens the 395 nm light lit up different parts of the body, like the hairs in the *Solpugema hostilis* specimen, resulting in a more detailed picture than the one with the 365 nm exposure. The reason for the difference in UV fluorescence between tissues is probably due to the molecular composition of it [11]. It is clear that it is difficult to predict which part of a specimen or which specimen will react to UV exposure. Considering the costs of the light bulbs, it is wise to add both of them to the digitization focus stacking equipment, to generate quality 2D+ images. For some specimens it will be necessary to make multiple pictures with illumination of different UV wavelengths to receive the maximum of information even if this would take more time to digitize a specimen. It might be possible, though, that using other light sources which emit UV of 395 nm with a more white light will reduce exposure time too. Generally most specimens will show equal amount of details whether exposed to 365 nm or 395 nm and because of the faster process the 365 nm lights are prefered when taking lots of UV pictures. The tests also proved out that only 2 lamps are sufficient. But sometimes the gain in detail is enough to justify the extra time needed when using the 395 nm lights. Of course not all species do react equally to UV light exposure ([10], [12]) and even within species variation exists ([2], [23], [24]). Some will show no reaction at all, while others will only partially lit up. In case of the latter it would be possible to combine UV light and flash exposure at the same time. However the tests reveal, that the parts that do lit up in UV light are sharper than in the combination picture. So it is advised to make 2 separate pictures instead.

The test performed with the yellow filter proved that is helps to remove lots of the blue emitted light. It also gives the picture more contrast which enhances several areas.

The most striking results were seen when dark specimens, like the *Pandinus imperator*, lit up under UV exposure. Therefore, taking UV pictures of dark specimens that fluoresce, is very useful as the gain in information is massive. The pictures of the low contrast Epimeria specimen show a bit more contrast than the normal flash exposed one. UV light might be of great aid in providing contrast when photographing uniformly colored specimens or specimens of which the color has faded due to the preservation process. It might also help to shed a new light on groups with cryptic species complexes. We know that not all specimens show fluorescence, but it is still possible they show a reaction to the UV exposure which isn’t in the visible spectrum. Canon (pers. comm.) notified that the lenses used do not have built-in UV filters. So to understand the real influence of UV, removing the internal filter and adding a UV pass filter (cut-off filter) might give great opportunities.

Most of the details which are visible with the combination of UV photography and focus stacking are difficult to see when using standard macroscopes. To see these details, SEM (Scanning Electron Microscope) images are required. However to obtain SEM image, most of the time the specimen has to be small, dried and gold covered. This makes the entire process time consuming, costly and eventually will destroy the natural state of specimen. The combination of UV photography and focus stacking can be an alternative for the larger specimens as this is a non invasive technique and can be tested quickly. Given the results of this study, the very low cost of a UV light setup, the numerous specimens that might show fluorescence, the approach of combining focus stacking with UV exposure is something to consider in any serious digitization program or when describing new species. Now it would be extremely interesting to test also the infrared spectrum, to see if there is an added value. Perhaps in the future full multispectral setups will be the standard both for focus stacking setups and 3D recording systems.

## Conclusion

As showed by the plethora of pictures in the above tests, the combination of low cost UV exposure and a 2D+ digitization setup enables to make highly detailed images of many specimens which fluoresce. Even though the colors produced as a reaction to the UV light can be awkward, this can be overcome by adding filters or simply converting the image into grayscale. The benefit of the system is that it can be used for specimens across collection and preservation boundaries. It will be interesting to test whether picturing non fluorescing specimens with UV light exposure and a UV pass filter will reveal more details because of the shorter wave length of the UV light.

## Supporting Information

S1 Table Specimen information. All specimens used are part of the collection of the Royal Belgian Institute of Natural Sciences (RBINS) or the Royal Museum for Central Africa (RMCA). Specimen numbers can be found below.
(XLS)

S2 Table Figure information. An overview of the pictures in the manuscripts showing: which illumination and the number of lights is used; whether an external filter was applied or an internal one was removed; which, white balance setting and possible post-treatment is chosen; the preservation state of the specimen and the name of the specimen pictured.
(XLS)
